# Supplementary material for: Baby HABIT-ILE intervention: study protocol of a randomised controlled trial in infants aged 6–18 months with unilateral cerebral palsy
Source: BMJ Open. 2024 Feb 17;14(2):e078383. doi: 10.1136/bmjopen-2023-078383 (PMC10875549; doi:10.1136/bmjopen-2023-078383)
Supplement: Author's manuscript [file bmjopen-2023-078383.draft_revisions.pdf]

BMJ Open is committed to open peer review. As part of this commitment we make the peer review history of every article we publish publicly available.

When an article is published we post the peer reviewers' comments and the authors' responses online. We also post the versions of the paper that were used during peer review. These are the versions that the peer review comments apply to.

The versions of the paper that follow are the versions that were submitted during the peer review process. They are not the versions of record or the final published versions. They should not be cited or distributed as the published version of this manuscript.

BMJ Open is an open access journal and the full, final, typeset and author-corrected version of record of the manuscript is available on our site with no access controls, subscription charges or pay-per-view fees (http://bmjopen.bmj.com).

If you have any questions on BMJ Open's open peer review process please email info.bmjopen@bmj.com

BMJ Open

Baby HABIT-ILE intervention: study protocol of a randomized controlled trial in infants aged 6-18 months with unilateral cerebral palsy

Journal:	*BMJ Open*
Manuscript ID	bmjopen-2023-078383
Article Type:	Protocol
Date Submitted by the Author:	01-Aug-2023

Complete List of Authors:	Carton de Tournai, Astrid; Université catholique de Louvain, Institute of Neuroscience<br>Herman, Enimie; Université catholique de Louvain, Institute of Neuroscience<br>Gathy, Estelle; Université catholique de Louvain, Institute of Neuroscience; CHU UCL Namur, Neurology Department, Stroke Unit/Motor Learning Lab<br>Ebner-Karestinos, Daniela; Université catholique de Louvain, Institute of Neuroscience; Universidad Andrés Bello, Exercise and Rehabilitation Science Institute, School of Physical Therapy, Faculty of Rehabilitation Science<br>Araneda, Rodrigo; Université catholique de Louvain, Institute of Neuroscience; Universidad Andrés Bello, Exercise and Rehabilitation Science Institute, School of Physical Therapy, Faculty of Rehabilitation Science<br>Dricot, Laurence; Université catholique de Louvain, Institute of Neuroscience; Université catholique de Louvain, Louvain Bionics<br>Macq, Benoît; Université catholique de Louvain, Institute of Neuroscience; Université catholique de Louvain, Institute of Information and Communication Technologies, Electronics and Applied Mathematics (ICTM)<br>Vandermeeren, Yves; CHU UCL Namur, Neurology Department, Stroke Unit/Motor Learning Lab; Université catholique de Louvain, Louvain Bionics<br>Bleyenheuft, Yannick; Université catholique de Louvain, Institute of Neuroscience; Université catholique de Louvain, Louvain Bionics

Keywords:	Randomized Controlled Trial, Paediatric neurology < NEUROLOGY, Rehabilitation medicine < INTERNAL MEDICINE, Neurological injury < NEUROLOGY, Developmental neurology & neurodisability < PAEDIATRICS, Magnetic resonance imaging < RADIOLOGY & IMAGING

SCHOLARONE™
Manuscripts

Baby HABIT-ILE intervention: study protocol of a randomized controlled trial in infants aged 6-18 months with unilateral cerebral palsy.

Carton de Tournai A[1,†] & Herman E[1,†], Gathy E[1,4], Ebner-Karestinos D[1,2], Araneda R[1,2], Dricot L[1,5], Macq B[1,3,5], Vandermeeren Y[4,5], Bleyenheuft Y[1,5].

[1] Institute of Neuroscience, Université catholique de Louvain, Brussels, Belgium.

[2] Exercise and Rehabilitation Science Institute, School of Physical Therapy, Faculty of Rehabilitation Science, Universidad Andres Bello, Santiago, Chile.

[3] Institute of Information and Communication Technologies, Electronics and Applied Mathematics (ICTM), Université catholique de Louvain, Louvain-la-Neuve, Belgium.

[4] Neurology Department, Stroke Unit/Motor Learning Lab., CHU UCL Namur, Site Godinne, Yvoir, Belgium

[5] Louvain Bionics, Université catholique de Louvain, Louvain-la-Neuve, Belgium

[†]These authors have contributed equally to this work and share first authorship

Corresponding authors:

Yannick Bleyenheuft, Institute of Neuroscience, Université catholique de Louvain, Avenue Mounier 53 box B1.53.04, 1200 Brussels, Belgium. yannick.bleyenheuft@uclouvain.be

For peer review only

ABSTRACT

Introduction: Research using animal models suggests that intensive motor skill training in infants under two years old with cerebral palsy (CP) may significantly reduce, or even prevent, maladaptive neuroplastic changes following brain injury. However, the effects of such interventions to tentatively prevent secondary neurological damages have never been assessed in infants with CP. This study aims to determine the effect of the baby Hand and Arm Bimanual Intensive Therapy-Including Lower Extremities (baby HABIT-ILE) in infants with unilateral CP, compared to a control intervention.

Methods and analysis: This randomized controlled trial will include 48 infants with unilateral CP aged (corrected if preterm) 6 to 18 months at the first assessment. They will be paired by age and by etiology of the CP, and randomized into 2 groups (immediate and delayed). Assessments will be performed at baseline and at 1 month, 3 months and 6 months after baseline. The immediate group will receive 50 hours of baby HABIT-ILE intervention over 2 weeks, between first and second assessment, while the delayed group will continue their usual activities. This last group will receive baby HABIT-ILE intervention after the 3 months assessment. Primary outcome will be the Mini-Assisting Hand Assessment. Secondary outcomes will include behavioral assessments for gross and fine motricity, visual – cognitive – language abilities as well as magnetic resonance imaging and kinematics measures. Moreover, parents will determine and score child-relevant goals and fill out questionnaires of participation, daily activities and mobility.

Ethics and dissemination: Full ethical approval has been obtained by the *Comité d'éthique Hospitalo-Facultaire/Université catholique de Louvain*, Brussels (2013/01MAR/069 B403201316810g). The recommendations of the ethical board and the Belgian law of May 7th 2004 concerning human experiments will be followed. Parents will sign a written informed consent ahead of participation. Findings will be published in peer-reviewed journals and conference presentations.

Trial registration number: NCT04698395. Registered on the International Clinical Trials Registry Platform (ICTRP) on December 2, 2020 and NIH Clinical Trials Registry on January 6, 2021.

URL of trial registry record: https://clinicaltrials.gov/ct2/show/NCT04698395?term=bleyenheuft&draw=1&rank=7

For peer review only

Strengths and limitations of this study:

- This study is a randomized controlled trial assessing motor and non-motor changes after an intensive intervention of motor activities in infants with unilateral cerebral palsy (CP) aged 6 to 18 months (corrected if preterm).
- This study will be the first RCT to apply a bimanual and locomotor intervention in this age group and to assess neuroplastic changes after an intervention in infants with unilateral CP.
- The primary outcome (Mini-Assisting Hand Assessment) and the secondary motor outcome (Gross Motor Function Measure-66) will be videotaped and blindly scored.
- Recruiting 24 pairs of infants in a narrow age range who fit our inclusion criteria such as an early diagnosis of unilateral CP might be a challenge.

Keywords: cerebral palsy, infants, intensive rehabilitation, neuroplasticity, HABIT-ILE, randomized controlled trial.

Word count: 3731

For peer review only

For peer review only - http://bmjopen.bmj.com/site/about/guidelines.xhtml

Introduction

Cerebral palsy (CP), the most prevalent pediatric motor disability, is a group of disorders of movement and posture due to non-progressive brain damage in the developing brain occurring in 1 out of 500 neonates (1). Depending on the location of the brain damage, children can present motor impairments (2), as well as associated symptoms including sensory, cognitive and visuo-spatial impairments (1). These impairments generally lead to highly variable limitations both in gross motor function and manual abilities, which may greatly impair the future execution of many activities of daily living (ADL; such as dressing, eating, toileting ...), affecting both the lifelong autonomy and the quality of life of these children (3–5).

As the brain damage is non-evolutive, the main focus of management of children with CP is currently the treatment of the symptoms, most of the time through neurorehabilitation based on neurodevelopmental approaches, usually a few hours a week including a mix of stretching and movements manually guided towards “normality” (6). However, the efficiency of these approaches is debated (6,7). In contrast, there is strong evidence that more recent intensive interventions based on motor skill learning principles are more efficient in inducing functional and neuroplastic changes, across age groups and disability levels, compared to traditional non-intensive neurorehabilitation (7–13).

The intensive interventions are organized as blocks of training using motor learning concepts (practice specificity, context of learning, feedback, etc)(14), aiming to elicit practice-induced brain changes (neuroplasticity) arising from repetition, increasing movement complexity, motivation and reward (15,16). Though efficient to improve motor abilities, these interventions remain limited to the mere treatment of symptoms.

Research using animal models (17,18) suggests that intensive motor skill training in infants with CP may significantly reduce or even prevent maladaptive neuroplastic changes

For peer review only - http://bmjopen.bmj.com/site/about/guidelines.xhtml

consecutive to the brain lesion, potentially addressing some of the root causes of CP. More specifically, the initial brain damage induces a cascade of secondary neuroplastic damages that account for the motor disability: chronic inflammation (19–21), alterations in the organization of the corticospinal tract (CST) (22,23) and underdeveloped CST projections at the spinal level (24,25). Moreover, due to the perception-action loop, the resulting motor deficit may in turn affect the developmental course of other functions such as attentional, visual and spatial cognition (26).

Only a few motor intensive intervention trials have been carried out in young children under 3 years old (27). These studies have focused on children with unilateral CP and evaluated modified forms of Constraint-Induced Movement Therapy (CIMT) (28–32) and environmental enrichment intervention (33). These studies have provided moderate support in favor of CIMT and enrichment intervention. Higher daily intensity of training (30,31) thereby seems to induce larger effects than lower daily intensity training (28,29,32). Even though lower extremities are commonly affected in children with CP, CIMT focuses solely on the upper extremities (28–32). Moreover, none of these studies have investigated the possible impact of these interventions on organization and projections of the CST, as well as on non-motor functions.

Hand and Arm Bimanual Intensive Therapy Including Lower Extremities (HABIT-ILE), developed by Bleyenheuft and Gordon (2014), is an intensive intervention based on motor skill learning principles which focuses on a bimanual use associated to postural and/or gross motor stimulation (10). Its efficiency was demonstrated for both upper and lower extremities in children with unilateral (8,34) and bilateral CP (9) from 1 to 18 years old. An impact has been observed on motor function, visuospatial abilities and improvement of brain structure in children (35). Such an intervention has never been attempted in infants aged between 6 to 18 months with CP to tentatively prevent secondary neurological damages or developmental trajectories.

For peer review only - http://bmjopen.bmj.com/site/about/guidelines.xhtml

Aims and hypotheses

This study aims to demonstrate the effectiveness and feasibility of an intensive motor skill learning intervention (baby HABIT-ILE) in preventing secondary damage and in inducing changes in motor and non-motor functions in children with unilateral CP, compared to a control intervention (usual activities). We hypothesize that the baby HABIT-ILE intervention will induce greater improvements in the immediate group compared to usual activities of the delayed group. Moreover, possible differences related to therapy onset will be observed, with a potential disadvantage for the delayed group due to the influence of a three months-lag in receiving intensive intervention during their specific window of opportunity. A randomized controlled trial (RCT) was designed to verify our hypothesis immediately after 2 weeks of therapy and at 3 months follow up. The results, scored by experts for the tests and by parents for the questionnaires, will be correlated with magnetic resonance imagery (MRI) changes.

Methods and analysis

Study design

This RCT will follow a two-parallel group design, involving forty-eight infants with unilateral CP between 6 and 18 months (corrected age). Assessments will be performed at the Institute of Neuroscience of the Université catholique de Louvain (UCLouvain) in Brussels, Belgium, at 3 intervals: baseline (T0), 1 month after baseline (T1) and 3 months after baseline (T2). The between-group comparisons will be carried out between T0 and T1 and between T0 and T2. The delayed group will receive after the third assessment the baby HABIT-ILE intervention. A supplementary assessment 6 months after baseline (T3) will allow investigating the influence of the onset of therapy and persistence of the therapeutic effects on the long term. At each assessment time, participants will follow motor and non-motor-, functionals skills-, participation-, kinematics- and neuroimaging assessments (see Fig.1).

For peer review only - http://bmjopen.bmj.com/site/about/guidelines.xhtml

The CONSORT statement for non-pharmaceutical and pragmatic trials will be used for the structure of this study and the SPIRIT guidelines were used for the writing of this protocol. The study will be conducted from February 2021 to December 2023.

Recruitment, inclusion and exclusion criteria

Forty-eight infants will be included. They will be recruited from rehabilitation centers of Belgium. Spontaneous applications from parents will also be considered.

To be eligible for inclusion, infants must be:

- ✓ Aged (corrected age if preterm birth) 6 to 18 months at the first assessment;
- ✓ Diagnosed with unilateral CP or suspicion because of asymmetry or at risk of developing unilateral CP;
- ✓ Able to follow instructions and complete testing according to the age.

Infants will be excluded if they have:

- ☒ Uncontrolled seizures;
- ☒ Botulinum toxin injections, orthopedic surgery or specific intensive therapy within 6 months before and until the end of the study;
- ☒ Implants or other metallic devices that are contraindications to MRI;
- ☒ Severe visual or cognitive impairments preventing the child from being interested in simple games.

Sample size calculation

The sample size calculation was performed based on the Assisting Hand Assessment (AHA(36)) score of a HABIT-ILE pilot study performed in 10 infants with unilateral CP (34). In this pilot study, toddlers with unilateral CP (1 to 4 years old) encountered an intensive intervention of 50 hours over 2 weeks, as in the present study. A mean improvement of 8,2 AHA units (standard deviation (SD)=5.9 AHA units) after the intensive therapy was reported.

For peer review only - http://bmjopen.bmj.com/site/about/guidelines.xhtml

Thus, our hypothesis is an incremental improvement of 1 SD between the immediate group and the delayed group at second and third assessments (between group difference =7,2, SD=5,9). With α=0.05 and a 1-β=0.9, a sample size of 15 participants per group is required. Considering potential dropouts and data loss in MRI, 24 participants will be included in each group (48 in total).

Randomization process

A pair-matched randomization will be used. Group allocation to either immediate or delayed condition will be randomly determined within each pair using an online computer-based randomization sequence (37). Children will be matched regarding age (± 4 months) and etiology of CP (brain malformation / periventricular white matter lesion / grey matter lesion; following Krägeloh-Mann's classification (2)) before the baseline assessment and collection of informed consent. If possible, children will also be matched considering the affected hemisphere (right/left).

Blinding procedure

The participants and interventionists (therapists) will be blind to the group allocation. Study allocation codes will be used for analyses and all data will be anonymized. The Mini-AHA and the Gross Motor Function Measure-66 (GMFM-66) will be videotaped and blindly scored by external accredited/experienced raters unaware of group allocation or assessment time. Anonymized data will be stored in the Research Electronic Data Capture (REDCap) register which is hosted at the UCLouvain. Once the data collection is over, anonymized data will be analyzed.

Study Interventions

Control intervention: Usual activities

For peer review only - http://bmjopen.bmj.com/site/about/guidelines.xhtml

Usual activities have been chosen as control intervention since spontaneous activity of infants is oriented towards the discovery of their environment with motor activities in a nursery reaching around 5 hours per day (38).The infants allocated to the delayed group will continue, between the first and the second assessments, their usual activities including nursery and ongoing therapy, generally a few hours of neurodevelopmental therapy per week. Wrist movement sensors will record the amount of motor activity during 5 days, 5 hours a day, to document the percentage of total time spent in movement during the control intervention.

Treatment intervention: baby HABIT-ILE

The intensive motor skill training consists of an adaptation of HABIT-ILE (8–10). This intervention, which was developed initially for children with CP over 6 years old, is carried out in a camp setting, with structured tasks of increasing motor difficulty and functional activities that require the use of both hands whilst sustaining postural activity or activities of the lower extremities. The therapy, using games and functional activities, is delivered in a child-friendly environment. The tasks are chosen according to individualized functional goals previously defined by the parents (e.g. drinking or sitting autonomously, turning the pages of a book, moving around). Although the basic principles of HABIT-ILE will be followed (10), the toys, tasks and activities will be adapted to the age of the infants. The duration of activities as well as lower extremities/postural requests will also be adapted to each infant depending on his/her functional goals and individual characteristics (motor and attentional level, for instance). Twelve infants will undergo rehabilitation at the same time during one intensive camp of 2 weeks (4 camps in total); each infant will have a full-time therapist dedicated to his/her treatment. The monitoring of the guidelines and the fidelity to the treatment principles will be ensured throughout the whole intervention by experienced HABIT-ILE supervisors. As 50 to 60 hours of intensive therapy are needed to induce long-term motor changes (39,40), this

For peer review only

project, in agreement with the usual motor activity time (38) and the need for infants to rest, will include 5 hours per day of therapy with the same therapist for a total of 50 hours (5days/week). HABIT-ILE for infants has been tested in a pilot study which has confirmed previous evidence that this therapy intensity is both practically feasible and efficient for children at that age (34). Infants will follow the intervention during 3 hours in the morning (with a short break for those who need a morning nap), they will subsequently have 2 hours of nap-break around noon, and will complete 2 additional hours of intervention in the afternoon.

Data collection and data management

An "assessment log sheet" including patient code, date of assessment, test procedure, mood of the child and context during the data acquisition will be used at each assessment. This information will be collected on REDCap (41,42) register. All children (immediate and delayed groups) will be seen at the 4 assessments times. At each assessment time, children will complete all the assessments listed below, except the ophthalmological assessment which is only carried out once.

Outcomes

Outcomes of the study were chosen to cover the 3 levels of the International Classification of Functioning, Disability and Health: Children & Youth version (ICF-CY)(43). A daily logbook filled by the interventionists during the whole therapy time will be used to examine the feasibility of the intervention. Interventionists will write the time and the type of activities for each participant during the whole camp duration.

Primary outcome

The Mini-AHA, as primary outcome, measures the use of the more affected hand collaboratively with the less affected hand in bimanual activities. This videotaped test targets the activity domain of the ICF-CY and uses Rasch model analysis. The score is expressed on a

For peer review only - http://bmjopen.bmj.com/site/about/guidelines.xhtml

logit based AHA-unit scale ranging from 0 to 100 (the higher the score, the better the ability). This assessment is responsive, reliable, and valid for use in children with unilateral CP (44).

Secondary outcomes

These outcomes concern the three levels of the ICF-CY. First, the body functions and structures will be studied through ophthalmological and neuroimaging assessments. Second, the activity level of the children will be assessed with the GMFM-66 (45), the Bayley Scales of Infant and toddler Development-Third edition (BSID-III) (46), the kinematics assessment (Vicon 3D motion system (47)), the kinetics assessment (Xsens-dot movement sensors (48)) and the visuospatial battery “Batterie d'évaluation du Jeune Enfant” (BAJE) (49). Moreover, questionnaires including the Pediatric Evaluation of Disability Inventory - Computer Adaptive Test (PEDI-CAT) (50,51) and the Canadian Occupational Performance Measure (COPM) (52) will assess both activity and participation levels. Third, the Young Children's Participation and Environment Measure (YC-PEM) (53), for its part, will only assess the participation level.

Body functions and structures assessments

To determine neuroplastic changes, MRI datasets will be acquired. Those assessments will take place in the Saint-Luc University Hospital in Brussels using a 3 Tesla MRI (SIGNA™ Premier, General Electric), equipped with a 48-channel head coil. The MRI procedure will consist in night scanning during natural, non-sedated sleep. Infants will undergo a standardized, gradual, adaptation home program to accustom them to the specific conditions of the MRI environment (wearing earplugs and careful examination of each infant's sleep routines (54)). Adapted transfer material allowing the infant to fall asleep in a child-friendly environment in the MRI service and to be transferred asleep in the machine will be used. The total time of MRI is estimated to 35 minutes. MRI changes will be measured with anatomical imaging (T1, T2 and multi-shell diffusion sequences) and functional imaging (resting-state sequence). More

For peer review only

precisely, our analyses will include the classical diffusion tensor imaging (DTI) indicators of fractional anisotropy, radial diffusivity, axial diffusivity, and mean diffusivity (55,56). In addition, new advanced model-based microstructural features based on angular weighting will be used to investigate microscopic level of training-induced changes (57,58). At macroscopic level, tractography will be used to highlight potential changes in CST and optic radiation organization (59,60).

All infants will encounter an ophthalmological assessment at the last assessment (T3) by ophthalmologists from the Saint-Luc University Hospital, Brussels. A standard battery of ophthalmological tests for infants will be used (Hirshberg(61), Gracis biprism (62), Lang II(63,64), Cover Test (65),ocular motility, Cardiff test (66), empty refraction, refraction under cycloplegia, dilated fundus, etc). This assessment will allow to exclude visual impairments of peripheral origin, to document visual impairments and to describe the baseline of the ophthalmological condition of these infants.

Activity assessments

Gross motor function will be measured using the GMFM-66 (45). This tool is a clinical assessment for children with CP allowing the measure of changes in gross motor function over time or following an intervention. The items cover a large spectrum of activities divided into 5 domains: 'lying and rolling', 'sitting', 'crawling and kneeling', 'standing', and 'walking, running and jumping'. This test will be videotaped and uses Rasch model analysis. The score is expressed on percentage of logits. This test is responsive (67), reliable and valid for infants with CP (68).

The BSID-III (46) will be used to assess cognitive, motor and language development. The cognitive scale includes attention and habituation tasks, problem solving, play tasks, object assembly, concept grouping and memory tasks. The motor scale, divided into two subscales includes assessments of gross and fine motor skills such as grasping, sitting, stacking blocks

For peer review only - http://bmjopen.bmj.com/site/about/guidelines.xhtml

and climbing stairs. Finally, the language scale, also divided into two subscales (receptive and expressive communication) includes understanding and expression of language (recognition of objects and people, following directions, naming and recognizing objects and pictures, etc). Raw scores of the items are converted to scaled scores and composite scores (mean 100, SD 15) ranging from 40 to 160 (higher scores indicate better performance). This assessment has been shown to be reliable and valid in young children with CP (69).

Kinematics assessments will be used to determine qualitative changes in upper extremities movement characteristics. Markers will be positioned on the head (using a headband), acromions, elbows and wrists of the child. Children will sit on an adapted chair, in front of a table. The first task will consist of a unimanual reaching of a toy, with the less-affected hand and the more-affected hand, separately. The second task will be bimanual: children will be asked to tap 2 cymbals against each other. Through a 3D motion system (Vicon Motion Systems, Oxford, UK (47)) the straightness (percentage of upper extremities trajectory during a reaching task) and smoothness (variability of movement during a reaching task) of the upper extremities will be studied for each task. Also, the time of activity (time from onset to end of the task in seconds) during a reaching task will be analysed.

Quantitative changes of physical activity will also be measured with two movement sensors (Xsens-Dot (48)), placed on each wrist during 5 hours a day for 5 days at home and during the camp duration. The percentage of total time spent in movement (i.e. crawling, walking and running) will be measured and calculated in terms of the changes in the acceleration (m/s^2).

In addition, the visuospatial battery BAJE (49) will be used to assess changes in visuo-spatial attention and visuo-attentional abilities (70). The BAJE is an adaptation of the Evaluation of Visuo-spatial Abilities (EVA) (70–72) which is designed to test visuospatial and visuo-attentional abilities of children from 3 months of age, developed and validated by Sylvie Chokron's team. This normalized and standardized battery includes simple tasks such as

For peer review only - http://bmjopen.bmj.com/site/about/guidelines.xhtml

photomotor reflex and blink reflex at threat, visual fixation, light detection, visual pursuit, visual filed/close visual field, visuo-motor coordination, … In this study, through three different tasks of this battery (visuo-motor coordination, visual field and eye pursuit), we will evaluate the presence and severity of the most common cerebral visual impairments (visual field defect, visuo-attentional, visuospatial and visuo-motor deficits).

The 'daily activities' and 'mobility' domains of the PEDI-CAT will be used to evaluate the capacity of upper and lower extremities of children with CP during specific activities (50). The scores are computed through the PEDI-CAT software (scores reported in scaled scores). This questionnaire is sensitive for children with CP (73).

The COPM (52), a semi-structured interview performed by a trained examiner, will be used to define the therapeutic goals and to quantify the child's performance (1 to 10 scale) in relation to these goals, as well as the satisfaction (1 to 10 scale) of the parents regarding their child's achievement. Total score is the average of the scores for performance and satisfaction separately.

Participation assessments

Participation will be evaluated using the PEDI-CAT(74), the COPM (52) and the YC-PEM (53). Based on different children's activities, the YC-PEM is a parents-filled questionnaire evaluating the level of participation and the quality of the environment across three settings: home, daycare/preschool and community. For each type of activity, caregivers assess multiple dimensions of the child's participation: frequency, level of involvement, caregiver's percent desire for change and perceived impact of environmental support. It has a good intra-rater and inter-rater reliability (53).

Statistical analysis

For peer review only - http://bmjopen.bmj.com/site/about/guidelines.xhtml

Statistical analysis will be performed on an intention-to-treat basis to account for data lost due to withdrawal. Analysis of covariance (ANCOVA) between groups measurements (or non-parametric equivalent when normality and homoscedasticity are not met) will be used for the primary outcome, the Mini-AHA, and the secondary outcomes. Significant differences will be statistically set at $\alpha = 0.05$.

Ethics and dissemination

Full ethical approval has been obtained for this study protocol by the Comité d'éthique Hospitalo-Facultaire/UCLouvain, Brussels (reference number: 2013/01MAR/069 B403201316810g). An information letter will be provided to the parents. They will be asked to sign a consent form if they comply to their child's participation. The parents will be informed during the recruitment process of their right to withdraw their child at any time without prejudice. All data collected will be anonymous and will be stored by the UCLouvain. Findings from this study will be published in peer-reviewed scientific journals and conference presentations. This study has been registered on NIH Clinical Trials Registry on January, 6 2021(NCT04698395 https://clinicaltrials.gov/ct2/show/NCT04698395?term=bleyenheuft&draw=1&rank=7).

Patient and public involvement

Patients and the public were not involved in the design, or conduct, or reporting, or dissemination plans of this research.

Conclusion

This RCT has been designed to substantiate evidence of the efficacy of the baby HABIT-ILE intervention to provide functional and neuroplastic changes in infants with unilateral CP. By implementing the baby HABIT-ILE intervention very early in the child's development during a specific window of opportunity, we hope to prevent the onset of secondary damages and,

For peer review only - http://bmjopen.bmj.com/site/about/guidelines.xhtml

consequently, to potentiate the long-term motor abilities of the children and increase their autonomy.

Abbreviations

AHA: Assisting Hand Assessment; ANCOVA: Analysis of Covariance; BAJE: Batterie d'évaluation du jeune enfant ; BSID-III: Bayley Scale of Infant and toddler Development-Third edition; COPM: Canadian Occupational Performance Measure; CP: Cerebral Palsy; CST: Corticospinal Tract; DTI: Diffusion Tensor Imaging; GMFM-66: Gross Motor Function Measure-66; HABIT-ILE: Hand and Arm Bimanual Intensive Therapy Including Lower Extremities; MRI: Magnetic Resonance Imaging; PEDI-CAT: Pediatric Evaluation of Disability Inventory - Computer Adaptive Test; RCT: Randomized Controlled Trial; REDCap: Research Electronic Data Capture; UCLouvain: Université catholique de Louvain; YC-PEM: Young Children's Participation and Environment Measure

Author contributions

YB, LD, BM, YV contributed to study design and obtained funding for the research study. AC and EH first drafted this protocol. YB, RA, DEK, EH, AC and EG will be involved in the implementation of the therapy. EH, AC, EG, DEK and RA will recruit the participants and perform the assessments. YB, AC, EH, LD, DEK and RA will contribute to data analysis. All authors contributed to the writing of this manuscript and have critically reviewed and approved the final version.

Funding

This study is part of a larger study funded by the Fédération Wallonie-Bruxelles through the program "*Action de Recherche Concertée*" of the UCLouvain, Belgium. Partial grant specifically dedicated to the visual part has also been provided by the "*Fondation JED-*

For peer review only - http://bmjopen.bmj.com/site/about/guidelines.xhtml

Belgique". EH has a grant from the "*Fonds de la Recherche Scientifique - FNRS"*. The funding bodies have reviewed this protocol during the selection process, but after this initial point, have participated neither in the study design nor in data collection, analysis, interpretation data, nor in writing the manuscript.

Competing interests

The authors have no conflict of interest to disclose.

For peer review only

For peer review only - http://bmjopen.bmj.com/site/about/guidelines.xhtml

REFERENCES

1. Graham HK, Rosenbaum P, Paneth N, Dan B, Lin JP, Damiano DL, et al. Cerebral palsy. Nat Rev Dis Primers [Internet]. 2016/05/18. 2016;2:15082. Available from: https://www.nature.com/articles/nrdp201582

2. Krägeloh-Mann I, Horber V. The role of magnetic resonance imaging in elucidating the pathogenesis of cerebral palsy: a systematic review. Dev Med Child Neurol [Internet]. 2007/01/27. 2007;49(2):144–51. Available from: https://onlinelibrary.wiley.com/doi/10.1111/j.1469-8749.2007.00144.x

3. Colver A, Rapp M, Eisemann N, Ehlinger V, Thyen U, Dickinson HO, et al. Self-reported quality of life of adolescents with cerebral palsy: a cross-sectional and longitudinal analysis. Lancet [Internet]. 2015 Feb 21 [cited 2022 Nov 15];385(9969):705–16. Available from: https://pubmed.ncbi.nlm.nih.gov/25301503/

4. Dickinson HO, Parkinson KN, Ravens-Sieberer U, Schirripa G, Thyen U, Arnaud C, et al. Self-reported quality of life of 8-12-year-old children with cerebral palsy: a cross-sectional European study. Lancet [Internet]. 2007 Jun 30 [cited 2022 Nov 15];369(9580):2171–8. Available from: https://pubmed.ncbi.nlm.nih.gov/17604799/

5. Kerr C, Mcdowell B, Mcdonough S. The relationship between gross motor function and participation restriction in children with cerebral palsy: an exploratory analysis. Child Care Health Dev [Internet]. 2007 Jan [cited 2022 Nov 15];33(1):22–7. Available from: https://pubmed.ncbi.nlm.nih.gov/17181749/

6. Te Velde A, Morgan C, Finch-Edmondson M, McNamara L, McNamara M, Paton MCB, et al. Neurodevelopmental Therapy for Cerebral Palsy: A Meta-analysis. Pediatrics [Internet]. 2022 Jun 1 [cited 2023 Apr 18];149(6). Available from: https://pubmed.ncbi.nlm.nih.gov/35607928/

7. Novak I, Morgan C, Fahey M, Finch-Edmondson M, Galea C, Hines A, et al. State of the Evidence Traffic Lights 2019: Systematic Review of Interventions for Preventing and Treating Children with Cerebral Palsy. Curr Neurol Neurosci Rep [Internet]. 2020/02/23. 2020;20(2):3. Available from: https://www.ncbi.nlm.nih.gov/pmc/articles/PMC7035308/pdf/11910_2020_Article_1022.pdf

8. Bleyenheuft Y, Arnould C, Brandao MB, Bleyenheuft C, Gordon AM. Hand and Arm Bimanual Intensive Therapy Including Lower Extremity (HABIT-ILE) in Children With Unilateral Spastic Cerebral Palsy: A Randomized Trial. Neurorehabil Neural Repair [Internet]. 2014/12/21. 2015;29(7):645–57. Available from: https://journals.sagepub.com/doi/pdf/10.1177/1545968314562109

9. Bleyenheuft Y, Ebner-Karestinos D, Surana B, Paradis J, Sidiropoulos A, Renders A, et al. Intensive upper- and lower-extremity training for children with bilateral cerebral palsy: a quasi-randomized trial. Dev Med Child Neurol [Internet]. 2017 Jun 1 [cited 2022 Nov 3];59(6):625–33. Available from: https://pubmed.ncbi.nlm.nih.gov/28133725/

10. Bleyenheuft Y, Gordon AM. Hand-arm bimanual intensive therapy including lower extremities (HABIT-ILE) for children with cerebral palsy. Phys Occup Ther Pediatr [Internet]. 2014/10/02. 2014;34(4):390–403. Available from: https://www.tandfonline.com/doi/full/10.3109/01942638.2014.932884

For peer review only - http://bmjopen.bmj.com/site/about/guidelines.xhtml

11. Hatem SM, Saussez G, della Faille M, Prist V, Zhang X, Dispa D, et al. Rehabilitation of Motor Function after Stroke: A Multiple Systematic Review Focused on Techniques to Stimulate Upper Extremity Recovery. Front Hum Neurosci [Internet]. 2016 Sep 13 [cited 2022 Nov 17];10(SEP2016):442. Available from: http://www.ncbi.nlm.nih.gov/pubmed/27679565

12. Brandão MB, Mancini MC, Ferre CL, Figueiredo PRP, Oliveira RHS, Gonçalves SC, et al. Does Dosage Matter? A Pilot Study of Hand-Arm Bimanual Intensive Training (HABIT) Dose and Dosing Schedule in Children with Unilateral Cerebral Palsy. Phys Occup Ther Pediatr [Internet]. 2018 May 27 [cited 2023 Apr 18];38(3):227–42. Available from: https://pubmed.ncbi.nlm.nih.gov/29240518/

13. Hoare BJ, Wallen MA, Thorley MN, Jackman ML, Carey LM, Imms C. Constraint-induced movement therapy in children with unilateral cerebral palsy. Cochrane Database Syst Rev [Internet]. 2019 Apr 1 [cited 2023 Apr 18];4(4). Available from: https://pubmed.ncbi.nlm.nih.gov/30932166/

14. Schmidt RA, Lee TD, Winstein C, Wulf G, Zelaznik HN. Motor control and learning: A behavioral emphasis. Human kinetics; 2018.

15. Nudo RJ. Adaptive plasticity in motor cortex: implications for rehabilitation after brain injury. J Rehabil Med [Internet]. 2003 [cited 2023 Apr 18];(41 Suppl):7–10. Available from: https://pubmed.ncbi.nlm.nih.gov/12817650/

16. Kleim JA, Hogg TM, VandenBerg PM, Cooper NR, Bruneau R, Remple M. Cortical synaptogenesis and motor map reorganization occur during late, but not early, phase of motor skill learning. J Neurosci [Internet]. 2004 Jan 21 [cited 2023 Apr 18];24(3):628–33. Available from: https://pubmed.ncbi.nlm.nih.gov/14736848/

17. Friel KM, Chakrabarty S, Martin JH. Pathophysiological mechanisms of impaired limb use and repair strategies for motor systems after unilateral injury of the developing brain. Dev Med Child Neurol [Internet]. 2013 Nov [cited 2023 Apr 13];55 Suppl 4(SUPPL.4):27–31. Available from: https://pubmed.ncbi.nlm.nih.gov/24237276/

18. Friel KM, Drew T, Martin JH. Differential activity-dependent development of corticospinal control of movement and final limb position during visually guided locomotion. J Neurophysiol [Internet]. 2007 May [cited 2023 Apr 13];97(5):3396–406. Available from: https://pubmed.ncbi.nlm.nih.gov/17376849/

19. Baud O, Daire JL, Dalmaz Y, Fontaine RH, Krueger RC, Sebag G, et al. Gestational hypoxia induces white matter damage in neonatal rats: a new model of periventricular leukomalacia. Brain Pathol [Internet]. 2004/03/05. 2004;14(1):1–10. Available from: https://onlinelibrary.wiley.com/doi/abs/10.1111/j.1750-3639.2004.tb00492.x?sid=nlm%3Apubmed

20. Favrais G, van de Looij Y, Fleiss B, Ramanantsoa N, Bonnin P, Stoltenburg-Didinger G, et al. Systemic inflammation disrupts the developmental program of white matter. Ann Neurol [Internet]. 2011/07/29. 2011;70(4):550–65. Available from: https://onlinelibrary.wiley.com/doi/abs/10.1002/ana.22489

21. van Steenwinckel J, Schang AL, Sigaut S, Chhor V, Degos V, Hagberg H, et al. Brain damage of the preterm infant: new insights into the role of inflammation. Biochem Soc Trans [Internet]. 2014/03/22. 2014;42(2):557–63. Available from:

For peer review only - http://bmjopen.bmj.com/site/about/guidelines.xhtml

https://portlandpress.com/biochemsoctrans/article-abstract/42/2/557/66817/Brain-damage-of-the-preterm-infant-new-insights?redirectedFrom=fulltext

22. Eyre JA, Taylor JP, Villagra F, Smith M, Miller S. Evidence of activity-dependent withdrawal of corticospinal projections during human development. Neurology [Internet]. 2001 Nov 13 [cited 2022 Nov 3];57(9):1543–54. Available from: https://pubmed.ncbi.nlm.nih.gov/11706088/

23. Staudt M. Brain Plasticity Following Early Life Brain Injury: Insights From Neuroimaging. Vol. 34, Seminars in Perinatology. 2010. p. 87–92.

24. Martin JH, Lee SJ. Activity-dependent competition between developing corticospinal terminations. Neuroreport. 1999/08/10. 1999;10(11):2277–82.

25. Martin JH. The corticospinal system: from development to motor control. Neuroscientist [Internet]. 2005/03/05. 2005;11(2):161–73. Available from: https://journals.sagepub.com/doi/10.1177/1073858404270843?url_ver=Z39.88-2003&rfr_id=ori:rid:crossref.org&rfr_dat=cr_pub%3Dpubmed

26. Chokron S, Dutton GN. Impact of Cerebral Visual Impairments on Motor Skills: Implications for Developmental Coordination Disorders. Front Psychol [Internet]. 2016 Oct 4 [cited 2022 Nov 3];7(OCT). Available from: https://pubmed.ncbi.nlm.nih.gov/27757087/

27. Damiano DL, Longo E. Early intervention evidence for infants with or at risk for cerebral palsy: an overview of systematic reviews. Dev Med Child Neurol [Internet]. 2021/04/08. 2021; Available from: https://onlinelibrary.wiley.com/doi/10.1111/dmcn.14855

28. Eliasson AC, Nordstrand L, Ek L, Lennartsson F, Sjöstrand L, Tedroff K, et al. The effectiveness of Baby-CIMT in infants younger than 12 months with clinical signs of unilateral-cerebral palsy; an explorative study with randomized design. Res Dev Disabil. 2017/11/28. 2018;72:191–201.

29. Eliasson AC, Shaw K, Berg E, Krumlinde-Sundholm L. An ecological approach of Constraint Induced Movement Therapy for 2-3-year-old children: a randomized control trial. Res Dev Disabil [Internet]. 2011 Nov [cited 2022 Nov 3];32(6):2820–8. Available from: https://pubmed.ncbi.nlm.nih.gov/21700416/

30. Taub E, Ramey SL, DeLuca S, Echols K. Efficacy of constraint-induced movement therapy for children with cerebral palsy with asymmetric motor impairment. Pediatrics [Internet]. 2004 Feb [cited 2022 Nov 3];113(2):305–12. Available from: https://pubmed.ncbi.nlm.nih.gov/14754942/

31. Deluca SC, Case-Smith J, Stevenson R, Ramey SL. Constraint-induced movement therapy (CIMT) for young children with cerebral palsy: effects of therapeutic dosage. J Pediatr Rehabil Med [Internet]. 2012 [cited 2022 Nov 3];5(2):133–42. Available from: https://pubmed.ncbi.nlm.nih.gov/22699104/

32. Nordstrand L, Holmefur M, Kits A, Eliasson AC. Improvements in bimanual hand function after baby-CIMT in two-year old children with unilateral cerebral palsy: A retrospective study. Res Dev Disabil. 2015/06/24. 2015;41–42:86–93.

For peer review only - http://bmjopen.bmj.com/site/about/guidelines.xhtml

33. Morgan C, Novak I, Dale RC, Guzzetta A, Badawi N. Single blind randomised controlled trial of GAME (Goals - Activity - Motor Enrichment) in infants at high risk of cerebral palsy. Res Dev Disabil. 2016/05/11. 2016;55:256–67.

34. Araneda R, Klöcker A, Ebner-Karestinos D, Sogbossi ES, Renders A, Saussez G, et al. Feasibility and effectiveness of HABIT-ILE in children aged 1 to 4 years with cerebral palsy: A pilot study. Ann Phys Rehabil Med. 2021;64(3):3–6.

35. Bleyenheuft Y, Dricot L, Ebner-Karestinos D, Paradis J, Saussez G, Renders A, et al. Motor Skill Training May Restore Impaired Corticospinal Tract Fibers in Children With Cerebral Palsy. Neurorehabil Neural Repair [Internet]. 2020 Jun 1 [cited 2022 Nov 3];34(6):533–46. Available from: https://pubmed.ncbi.nlm.nih.gov/32407247/

36. Krumlinde-Sundholm L, Eliasson AC. Development of the Assisting Hand Assessment: a Rasch-built measure intended for children with unilateral upper limb impairments. Scand J Occup Ther. 2003;10(1):16–26.

37. Research Randomizer [Internet]. Available from: https://randomizer.org/

38. Van Cauwenberghe E, Gubbels J, De Bourdeaudhuij I, Cardon G. Feasibility and validity of accelerometer measurements to assess physical activity in toddlers. Int J Behav Nutr Phys Act [Internet]. 2011 Jun 26 [cited 2023 Jan 5];8. Available from: https://pubmed.ncbi.nlm.nih.gov/21703004/

39. Brandão MB, Mancini MC, Ferre CL, Figueiredo PRP, Oliveira RHS, Gonçalves SC, et al. Does Dosage Matter? A Pilot Study of Hand-Arm Bimanual Intensive Training (HABIT) Dose and Dosing Schedule in Children with Unilateral Cerebral Palsy. Phys Occup Ther Pediatr [Internet]. 2018 May 27 [cited 2022 Nov 8];38(3):227–42. Available from: https://pubmed.ncbi.nlm.nih.gov/29240518/

40. Sakzewski L, Provan K, Ziviani J, Boyd RN. Comparison of dosage of intensive upper limb therapy for children with unilateral cerebral palsy: how big should the therapy pill be? Res Dev Disabil [Internet]. 2015 Feb 1 [cited 2022 Nov 8];37:9–16. Available from: https://pubmed.ncbi.nlm.nih.gov/25460215/

41. Harris PA, Taylor R, Minor BL, Elliott V, Fernandez M, O'Neal L, et al. The REDCap consortium: Building an international community of software platform partners. J Biomed Inform [Internet]. 2019 Jul 1 [cited 2022 Nov 9];95. Available from: https://pubmed.ncbi.nlm.nih.gov/31078660/

42. Harris PA, Taylor R, Thielke R, Payne J, Gonzalez N, Conde JG. Research electronic data capture (REDCap)--a metadata-driven methodology and workflow process for providing translational research informatics support. J Biomed Inform [Internet]. 2009 Apr [cited 2022 Nov 9];42(2):377–81. Available from: https://pubmed.ncbi.nlm.nih.gov/18929686/

43. Organization WH. International Classification of Functioning, Disability, and Health: Children & Youth Version: ICF-CY. World Health Organization; 2007.

44. Greaves S, Imms C, Dodd K, Krumlinde-Sundholm L. Development of the Mini-Assisting Hand Assessment: evidence for content and internal scale validity. Dev Med Child Neurol [Internet]. 2013/08/01. 2013;55(11):1030–7. Available from: https://onlinelibrary.wiley.com/doi/pdfdirect/10.1111/dmcn.12212?download=true

For peer review only - http://bmjopen.bmj.com/site/about/guidelines.xhtml

45. Russell DJ, Avery LM, Walter SD, Hanna SE, Bartlett DJ, Rosenbaum PL, et al. Development and validation of item sets to improve efficiency of administration of the 66-item Gross Motor Function Measure in children with cerebral palsy. Dev Med Child Neurol [Internet]. 2009/10/09. 2010;52(2):e48-54. Available from: https://onlinelibrary.wiley.com/doi/10.1111/j.1469-8749.2009.03481.x

46. Bayley N. Bayley-III: Bayley Scales of infant and toddler development. Giunti OS; 2009.

47. Merriaux P, Dupuis Y, Boutteau R, Vasseur P, Savatier X. A study of vicon system positioning performance. Sensors. 2017;17(7):1591.

48. Ribera D'alcala' E, Voerman JA, Konrath JM, Vydhyanathan A. Xsens DOT Wearable Sensor Platform White Paper. 2021;

49. Petkovic M, Chokron S, Fagard J. Visuo-manual coordination in preterm infants without neurological impairments. Res Dev Disabil [Internet]. 2016 Apr 1 [cited 2022 Nov 9];51–52:76–88. Available from: https://pubmed.ncbi.nlm.nih.gov/26812594/

50. Kramer JM, Liljenquist K, Coster WJ. Validity, reliability, and usability of the Pediatric Evaluation of Disability Inventory-Computer Adaptive Test for autism spectrum disorders. Dev Med Child Neurol [Internet]. 2015/06/25. 2016;58(3):255–61. Available from: https://onlinelibrary.wiley.com/doi/pdfdirect/10.1111/dmcn.12837?download=true

51. Thompson S V., Cech DJ, Cahill SM, Krzak JJ. Linking the Pediatric Evaluation of Disability Inventory-Computer Adaptive Test (PEDI-CAT) to the International Classification of Function. Pediatr Phys Ther [Internet]. 2018 Apr 1 [cited 2023 Apr 18];30(2):113–8. Available from: https://pubmed.ncbi.nlm.nih.gov/29498960/

52. Dedding C, Cardol M, Eyssen IC, Dekker J, Beelen A. Validity of the Canadian Occupational Performance Measure: a client-centred outcome measurement. Clin Rehabil. 2004/10/12. 2004;18(6):660–7.

53. Khetani MA, Graham JE, Davies PL, Law MC, Simeonsson RJ. Psychometric properties of the Young Children's Participation and Environment Measure. Arch Phys Med Rehabil. 2014/12/03. 2015;96(2):307–16.

54. Dean DC, Dirks H, O'Muircheartaigh J, Walker L, Jerskey BA, Lehman K, et al. Pediatric neuroimaging using magnetic resonance imaging during non-sedated sleep. Pediatr Radiol [Internet]. 2014 Jan [cited 2022 Nov 9];44(1):64–72. Available from: https://pubmed.ncbi.nlm.nih.gov/23917588/

55. Beaulieu C. The basis of anisotropic water diffusion in the nervous system - a technical review. NMR Biomed [Internet]. 2002 Nov [cited 2023 Apr 13];15(7–8):435–55. Available from: https://pubmed.ncbi.nlm.nih.gov/12489094/

56. Alexander AL, Lee JE, Lazar M, Field AS. Diffusion tensor imaging of the brain. Neurotherapeutics [Internet]. 2007 Jul [cited 2023 Apr 13];4(3):316–29. Available from: https://pubmed.ncbi.nlm.nih.gov/17599699/

57. Rensonnet G, Scherrer B, Girard G, Jankovski A, Warfield SK, Macq B, et al. Towards microstructure fingerprinting: Estimation of tissue properties from a dictionary of Monte Carlo diffusion MRI simulations. Neuroimage [Internet]. 2019 Jan 1 [cited 2023 Apr 13];184:964–80. Available from: https://pubmed.ncbi.nlm.nih.gov/30282007/

For peer review only - http://bmjopen.bmj.com/site/about/guidelines.xhtml

58. Scherrer B, Schwartzman A, Taquet M, Sahin M, Prabhu SP, Warfield SK. Characterizing brain tissue by assessment of the distribution of anisotropic microstructural environments in diffusion-compartment imaging (DIAMOND). Magn Reson Med [Internet]. 2016 Sep 1 [cited 2023 Apr 13];76(3):963–77. Available from: https://pubmed.ncbi.nlm.nih.gov/26362832/

59. Baek SO, Jang SH, Lee E, Kim S, Hah JO, Park YH, et al. CST recovery in pediatric hemiplegic patients: Diffusion tensor tractography study. Neurosci Lett [Internet]. 2013 Dec 17 [cited 2023 Apr 13];557 Pt B(PB):79–83. Available from: https://pubmed.ncbi.nlm.nih.gov/24176879/

60. Araneda R, Ebner-Karestinos D, Dricot L, Herman E, Hatem SM, Friel KM, et al. Impact of early brain lesions on the optic radiations in children with cerebral palsy. Front Neurosci [Internet]. 2022 Oct 5 [cited 2023 Apr 13];16. Available from: https://pubmed.ncbi.nlm.nih.gov/36278011/

61. Jones R, Eskridge JB. THE HIRSCHBERG TEST — A RE-EVALUATION*. Optometry and Vision Science [Internet]. 1970;47(2). Available from: https://journals.lww.com/optvissci/Fulltext/1970/02000/THE_HIRSCHBERG_TEST___A_RE_EVALUATION_.4.aspx

62. Paris V. [Screening and treatment of fixation dominance in primary microstrabismus]. Bull Soc Belge Ophtalmol [Internet]. 1992;243:45–53. Available from: http://europepmc.org/abstract/MED/1302153

63. Joseph L. A New Stereotest. Journal of Pediatric Ophthalmology & Strabismus [Internet]. 1983 Mar 1;20(2):72–4. Available from: https://doi.org/10.3928/0191-3913-19830301-08

64. Ohlsson J, Villarreal G, Sjöström A, Abrahamsson M, Sjöstrand J. Screening for amblyopia and strabismus with the Lang II stereo card. Acta Ophthalmol Scand [Internet]. 2002 Apr 1;80(2):163–6. Available from: https://doi.org/10.1034/j.1600-0420.2002.800208.x

65. PELI ELI, McCORMACK G. Dynamics of cover test eye movements. Optometry and Vision Science. 1983;60(8):712–24.

66. Adoh TO, Woodhouse JM. The Cardiff acuity test used for measuring visual acuity development in toddlers. Vision Res. 1994;34(4):555–60.

67. Vos-Vromans DC, Ketelaar M, Gorter JW. Responsiveness of evaluative measures for children with cerebral palsy: the Gross Motor Function Measure and the Pediatric Evaluation of Disability Inventory. Disabil Rehabil [Internet]. 2005/11/22. 2005;27(20):1245–52. Available from: https://www.tandfonline.com/doi/abs/10.1080/09638280500076178

68. Wei S, Su-Juan W, Yuan-Gui L, Hong Y, Xiu-Juan X, Xiao-Mei S. Reliability and Validity of the GMFM-66 in 0- to 3-Year-Old Children with Cerebral Palsy. Am J Phys Med Rehabil [Internet]. 2006/01/24. 2006 Feb;85(2):141–7. Available from: http://journals.lww.com/00002060-200602000-00006

69. Gill K, Osiovich A, Synnes A, J AA, Grunau RE, Miller SP, et al. Concurrent Validity of the Bayley-III and the Peabody Developmental Motor Scales-2 at 18 Months. Phys Occup Ther Pediatr [Internet]. 2018/12/14. 2019;39(5):514–24. Available from: https://www.tandfonline.com/doi/full/10.1080/01942638.2018.1546255

For peer review only

For peer review only - http://bmjopen.bmj.com/site/about/guidelines.xhtml

70. Chokron S. Evaluation of visuo-spatial abilities (EVA): a simple and rapid battery to screen for CVI in young children. Impairment of vision due to disorders of the visual brain in childhood: a practical approach. 2015;

71. Cavézian C, Vilayphonh M, Vasseur V, Caputo G, Laloum L, Chokron S. Ophthalmic disorder may affect visuo-attentional performance in childhood. Child Neuropsychology. 2013 May 1;19(3):292–312.

72. Cavézian C, Vilayphonh M, de Agostini M, Vasseur V, Watier L, Kazandjian S, et al. Assessment of visuo-attentional abilities in young children with or without visual disorder: toward a systematic screening in the general population. Res Dev Disabil [Internet]. 2010 Sep [cited 2022 Nov 9];31(5):1102–8. Available from: https://pubmed.ncbi.nlm.nih.gov/20413265/

73. Ko J. Sensitivity to functional improvements of GMFM-88, GMFM-66, and PEDI mobility scores in young children with cerebral palsy. Percept Mot Skills. 2014/08/26. 2014;119(1):305–19.

74. Scott K, Lewis J, Pan X, Heathcock J. Parent-Reported PEDI-CAT Mobility and Gross Motor Function in Infants With Cerebral Palsy. Pediatr Phys Ther [Internet]. 2021/06/05. 2021;33(3):156–61. Available from: https://www.ingentaconnect.com/content/wk/pep/2021/00000033/00000003/art00013;jsessionid=3kiit2ff2cua.x-ic-live-03

For peer review only

FIGURE

Figure 1: CONSORT Flowchart. CP, cerebral palsy; HABIT-ILE, Hand and Arm Bimanual Intensive Therapy Including Lower Extremities.

For peer review only

For peer review only - http://bmjopen.bmj.com/site/about/guidelines.xhtml

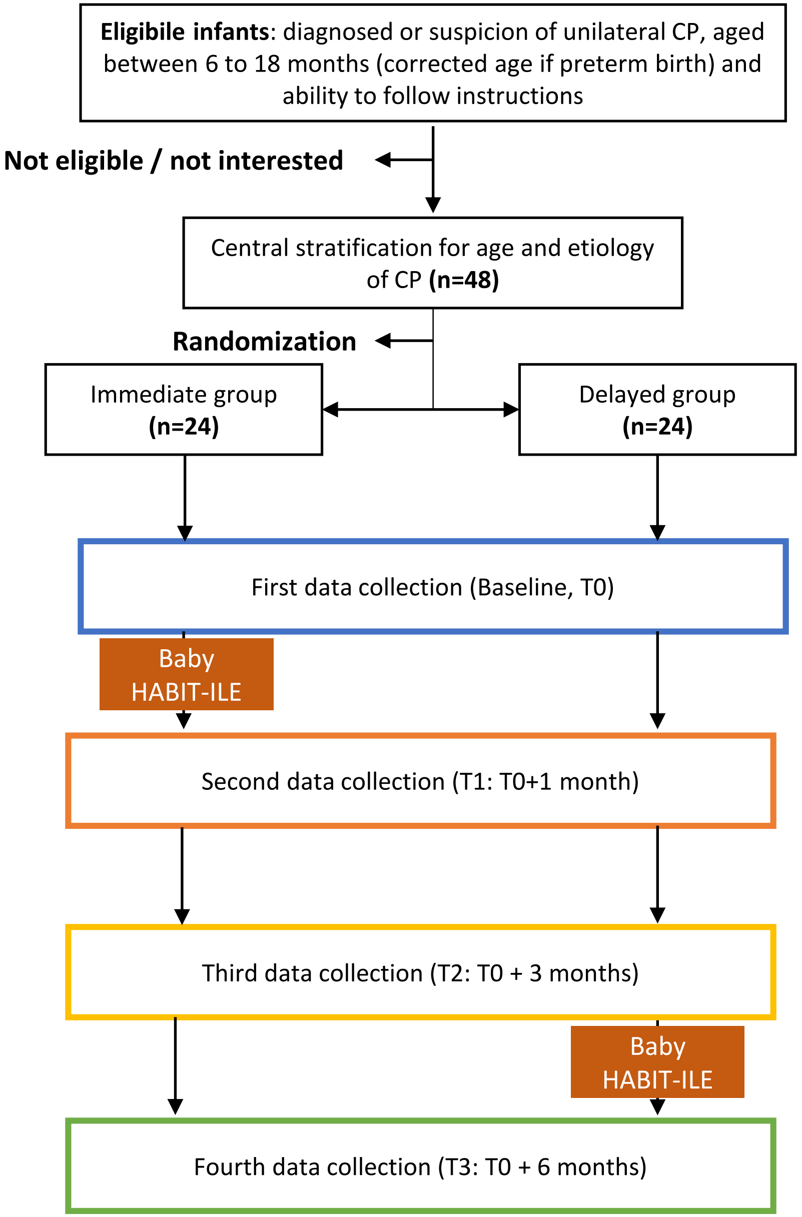

Assessments of T0, T1, T2 & T3

Body functions and structures

- Ophthalmological assessments (only once)
- Neuroimaging (MRI): anatomical imaging (T1, T2 and multi-shell diffusion) & functional imaging (resting-state)

Activity

- Mini-Assisting Hand Assessment (Mini-AHA; main outcome)
- Gross Motor Function Measure – 66 (GMFM-66)
- The Bayley Scales of Infant and toddler Development – Third Edition (BSID-III): Gross and fine motor, receptive and expressive communication & cognitive scales
- Kinematics of the upper extremity
- « Batterie d'évaluation du Jeune Enfant" (BAJE)
- The Pediatric Evaluation of Disability Inventory - computer adaptive test (PEDI-CAT)
- Canadian Occupational Performance Measure (COPM)

Participation

- The Pediatric Evaluation of Disability Inventory - computer adaptive test (PEDI-CAT)
- Young Children's Participation and Environment Measure (YCPEM)
- Canadian Occupational Performance Measure (COPM)

Reporting checklist for protocol of a clinical trial.

Based on the SPIRIT guidelines.

Instructions to authors

Complete this checklist by entering the page numbers from your manuscript where readers will find each of the items listed below.

Your article may not currently address all the items on the checklist. Please modify your text to include the missing information. If you are certain that an item does not apply, please write "n/a" and provide a short explanation.

Upload your completed checklist as an extra file when you submit to a journal.

In your methods section, say that you used the SPIRITreporting guidelines, and cite them as:

Chan A-W, Tetzlaff JM, Gøtzsche PC, Altman DG, Mann H, Berlin J, Dickersin K, Hróbjartsson A, Schulz KF, Parulekar WR, Krleža-Jerić K, Laupacis A, Moher D. SPIRIT 2013 Explanation and Elaboration: Guidance for protocols of clinical trials. BMJ. 2013;346:e7586

		Reporting Item	Page Number
**Administrative information**			
Title	#1	Descriptive title identifying the study design, population, interventions, and, if applicable, trial acronym	1
Trial registration	#2a	Trial identifier and registry name. If not yet registered, name of intended registry	2,15
Trial registration: data set	#2b	All items from the World Health Organization Trial Registration Data Set	n/a
Protocol version	#3	Date and version identifier	n/a
Funding	#4	Sources and types of financial, material, and other support	17
Roles and responsibilities: contributorship	#5a	Names, affiliations, and roles of protocol contributors	1,16,17

For peer review only

Roles and responsibilities: sponsor contact information	#5b	Name and contact information for the trial sponsor	n/a
Roles and responsibilities: sponsor and funder	#5c	Role of study sponsor and funders, if any, in study design; collection, management, analysis, and interpretation of data; writing of the report; and the decision to submit the report for publication, including whether they will have ultimate authority over any of these activities	17
Roles and responsibilities: committees	#5d	Composition, roles, and responsibilities of the coordinating centre, steering committee, endpoint adjudication committee, data management team, and other individuals or groups overseeing the trial, if applicable (see Item 21a for data monitoring committee)	n/a
**Introduction**			
Background and rationale	#6a	Description of research question and justification for undertaking the trial, including summary of relevant studies (published and unpublished) examining benefits and harms for each intervention	4,5
Background and rationale: choice of comparators	#6b	Explanation for choice of comparators	4,5
Objectives	#7	Specific objectives or hypotheses	6
Trial design	#8	Description of trial design including type of trial (eg, parallel group, crossover, factorial, single group), allocation ratio, and framework (eg, superiority, equivalence, non-inferiority, exploratory)	6
**Methods: Participants, interventions, and outcomes**			
Study setting	#9	Description of study settings (eg, community clinic, academic hospital) and list of countries where data will be collected. Reference to where list of study sites can be obtained	6
Eligibility criteria	#10	Inclusion and exclusion criteria for participants. If applicable, eligibility criteria for study centres and individuals who will	7

For peer review only

		perform the interventions (eg, surgeons, psychotherapists)	
Interventions: description	#11a	Interventions for each group with sufficient detail to allow replication, including how and when they will be administered	9, 10
Interventions: modifications	#11b	Criteria for discontinuing or modifying allocated interventions for a given trial participant (eg, drug dose change in response to harms, participant request, or improving / worsening disease)	n/a
Interventions: adherance	#11c	Strategies to improve adherence to intervention protocols, and any procedures for monitoring adherence (eg, drug tablet return; laboratory tests)	9,10
Interventions: concomitant care	#11d	Relevant concomitant care and interventions that are permitted or prohibited during the trial	n/a
Outcomes	#12	Primary, secondary, and other outcomes, including the specific measurement variable (eg, systolic blood pressure), analysis metric (eg, change from baseline, final value, time to event), method of aggregation (eg, median, proportion), and time point for each outcome. Explanation of the clinical relevance of chosen efficacy and harm outcomes is strongly recommended	10-15
Participant timeline	#13	Time schedule of enrolment, interventions (including any run-ins and washouts), assessments, and visits for participants. A schematic diagram is highly recommended (see Figure)	6
Sample size	#14	Estimated number of participants needed to achieve study objectives and how it was determined, including clinical and statistical assumptions supporting any sample size calculations	7,8
Recruitment	#15	Strategies for achieving adequate participant enrolment to reach target sample size	7
**Methods: Assignment of interventions (for controlled trials)**			
Allocation: sequence generation	#16a	Method of generating the allocation sequence (eg, computer-generated random numbers), and list of any factors for stratification. To reduce predictability of a random sequence, details of any planned restriction (eg, blocking) should be provided in a separate document that is unavailable to those who enrol participants or assign interventions	8

For peer review only

Allocation concealment mechanism	#16b	Mechanism of implementing the allocation sequence (eg, central telephone; sequentially numbered, opaque, sealed envelopes), describing any steps to conceal the sequence until interventions are assigned	n/a
Allocation: implementation	#16c	Who will generate the allocation sequence, who will enrol participants, and who will assign participants to interventions	8,17
Blinding (masking)	#17a	Who will be blinded after assignment to interventions (eg, trial participants, care providers, outcome assessors, data analysts), and how	8
Blinding (masking): emergency unblinding	#17b	If blinded, circumstances under which unblinding is permissible, and procedure for revealing a participant's allocated intervention during the trial	n/a
**Methods: Data collection, management, and analysis**			
Data collection plan	#18a	Plans for assessment and collection of outcome, baseline, and other trial data, including any related processes to promote data quality (eg, duplicate measurements, training of assessors) and a description of study instruments (eg, questionnaires, laboratory tests) along with their reliability and validity, if known. Reference to where data collection forms can be found, if not in the protocol	10
Data collection plan: retention	#18b	Plans to promote participant retention and complete follow-up, including list of any outcome data to be collected for participants who discontinue or deviate from intervention protocols	n/a
Data management	#19	Plans for data entry, coding, security, and storage, including any related processes to promote data quality (eg, double data entry; range checks for data values). Reference to where details of data management procedures can be found, if not in the protocol	10
Statistics: outcomes	#20a	Statistical methods for analysing primary and secondary outcomes. Reference to where other details of the statistical analysis plan can be found, if not in the protocol	15
Statistics: additional analyses	#20b	Methods for any additional analyses (eg, subgroup and adjusted analyses)	n/a

For peer review only

Statistics: analysis population and missing data	#20c	Definition of analysis population relating to protocol non-adherence (eg, as randomised analysis), and any statistical methods to handle missing data (eg, multiple imputation)	n/a
**Methods: Monitoring**			
Data monitoring: formal committee	#21a	Composition of data monitoring committee (DMC); summary of its role and reporting structure; statement of whether it is independent from the sponsor and competing interests; and reference to where further details about its charter can be found, if not in the protocol. Alternatively, an explanation of why a DMC is not needed	n/a
Data monitoring: interim analysis	#21b	Description of any interim analyses and stopping guidelines, including who will have access to these interim results and make the final decision to terminate the trial	n/a
Harms	#22	Plans for collecting, assessing, reporting, and managing solicited and spontaneously reported adverse events and other unintended effects of trial interventions or trial conduct	10
Auditing	#23	Frequency and procedures for auditing trial conduct, if any, and whether the process will be independent from investigators and the sponsor	n/a
**Ethics and dissemination**			
Research ethics approval	#24	Plans for seeking research ethics committee / institutional review board (REC / IRB) approval	15
Protocol amendments	#25	Plans for communicating important protocol modifications (eg, changes to eligibility criteria, outcomes, analyses) to relevant parties (eg, investigators, REC / IRBs, trial participants, trial registries, journals, regulators)	15
Consent or assent	#26a	Who will obtain informed consent or assent from potential trial participants or authorised surrogates, and how (see Item 32)	15
Consent or assent: ancillary studies	#26b	Additional consent provisions for collection and use of participant data and biological specimens in ancillary studies, if applicable	n/a
Confidentiality	#27	How personal information about potential and enrolled participants will be collected, shared, and maintained in order to	15

For peer review only

		protect confidentiality before, during, and after the trial	
Declaration of interests	#28	Financial and other competing interests for principal investigators for the overall trial and each study site	17
Data access	#29	Statement of who will have access to the final trial dataset, and disclosure of contractual agreements that limit such access for investigators	15
Ancillary and post trial care	#30	Provisions, if any, for ancillary and post-trial care, and for compensation to those who suffer harm from trial participation	n/a
Dissemination policy: trial results	#31a	Plans for investigators and sponsor to communicate trial results to participants, healthcare professionals, the public, and other relevant groups (eg, via publication, reporting in results databases, or other data sharing arrangements), including any publication restrictions	15
Dissemination policy: authorship	#31b	Authorship eligibility guidelines and any intended use of professional writers	n/a
Dissemination policy: reproducible research	#31c	Plans, if any, for granting public access to the full protocol, participant-level dataset, and statistical code	15
**Appendices**			
Informed consent materials	#32	Model consent form and other related documentation given to participants and authorised surrogates	appendix 1
Biological specimens	#33	Plans for collection, laboratory evaluation, and storage of biological specimens for genetic or molecular analysis in the current trial and for future use in ancillary studies, if applicable	n/a

The SPIRIT Explanation and Elaboration paper is distributed under the terms of the Creative Commons Attribution License CC-BY-NC. This checklist was completed on 17. July 2023 using https://www.goodreports.org/, a tool made by the EQUATOR Network in collaboration with Penelope.ai

For peer review only

BMJ Open

Baby HABIT-ILE intervention: study protocol of a randomized controlled trial in infants aged 6-18 months with unilateral cerebral palsy

Journal:	*BMJ Open*
Manuscript ID	bmjopen-2023-078383.R1
Article Type:	Protocol
Date Submitted by the Author:	26-Dec-2023
Complete List of Authors:	Carton de Tournai, Astrid; Université catholique de Louvain, Institute of Neuroscience<br>Herman, Enimie; Université catholique de Louvain, Institute of Neuroscience<br>Gathy, Estelle; Université catholique de Louvain, Institute of Neuroscience; CHU UCL Namur, Neurology Department, Stroke Unit/Motor Learning Lab<br>Ebner-Karestinos, Daniela; Université catholique de Louvain, Institute of Neuroscience; Universidad Andrés Bello, Exercise and Rehabilitation Science Institute, School of Physical Therapy, Faculty of Rehabilitation Science<br>Araneda, Rodrigo; Université catholique de Louvain, Institute of Neuroscience; Universidad Andrés Bello, Exercise and Rehabilitation Science Institute, School of Physical Therapy, Faculty of Rehabilitation Science<br>Dricot, Laurence; Université catholique de Louvain, Institute of Neuroscience; Université catholique de Louvain, Louvain Bionics<br>Macq, Benoît; Université catholique de Louvain, Institute of Neuroscience; Université catholique de Louvain, Institute of Information and Communication Technologies, Electronics and Applied Mathematics (ICTM)<br>Vandermeeren, Yves; CHU UCL Namur, Neurology Department, Stroke Unit/Motor Learning Lab; Université catholique de Louvain, Louvain Bionics<br>Bleyenheuft, Yannick; Université catholique de Louvain, Institute of Neuroscience; Université catholique de Louvain, Louvain Bionics
**Primary Subject Heading**:	Paediatrics
Secondary Subject Heading:	Neurology, Rehabilitation medicine
Keywords:	Randomized Controlled Trial, Paediatric neurology < NEUROLOGY, Rehabilitation medicine < INTERNAL MEDICINE, Neurological injury < NEUROLOGY, Developmental neurology & neurodisability < PAEDIATRICS, Magnetic resonance imaging < RADIOLOGY & IMAGING

For peer review only - http://bmjopen.bmj.com/site/about/guidelines.xhtml

Baby HABIT-ILE intervention: study protocol of a randomized controlled trial in infants aged 6-18 months with unilateral cerebral palsy.

Carton de Tournai A[1,†] & Herman E[1,†], Gathy E[1,4], Ebner-Karestinos D[1,2], Araneda R[1,2], Dricot L[1,5], Macq B[1,3,5], Vandermeeren Y[4,5], Bleyenheuft Y[1,5].

[1] Institute of Neuroscience, Université catholique de Louvain, Brussels, Belgium.
[2] Exercise and Rehabilitation Science Institute, School of Physical Therapy, Faculty of Rehabilitation Science, Universidad Andres Bello, Santiago, Chile.
[3] Institute of Information and Communication Technologies, Electronics and Applied Mathematics (ICTM), Université catholique de Louvain, Louvain-la-Neuve, Belgium.
[4] Neurology Department, Stroke Unit/Motor Learning Lab., CHU UCL Namur, Site Godinne, Yvoir, Belgium
[5] Louvain Bionics, Université catholique de Louvain, Louvain-la-Neuve, Belgium

[†]These authors have contributed equally to this work and share first authorship

Corresponding authors:

Yannick Bleyenheuft, Institute of Neuroscience, Université catholique de Louvain, Avenue Mounier 53 box B1.53.04, 1200 Brussels, Belgium. yannick.bleyenheuft@uclouvain.be

For peer review only - http://bmjopen.bmj.com/site/about/guidelines.xhtml

ABSTRACT

Introduction: Research using animal models suggests that intensive motor skill training in infants under two years old with cerebral palsy (CP) may significantly reduce, or even prevent, maladaptive neuroplastic changes following brain injury. However, the effects of such interventions to tentatively prevent secondary neurological damages have never been assessed in infants with CP. This study aims to determine the effect of the baby Hand and Arm Bimanual Intensive Therapy-Including Lower Extremities (baby HABIT-ILE) in infants with unilateral CP, compared to a control intervention.

Methods and analysis: This randomized controlled trial will include 48 infants with unilateral CP aged (corrected if preterm) 6 to 18 months at the first assessment. They will be paired by age and by etiology of the CP, and randomized into 2 groups (immediate and delayed). Assessments will be performed at baseline and at 1 month, 3 months and 6 months after baseline. The immediate group will receive 50 hours of baby HABIT-ILE intervention over 2 weeks, between first and second assessment, while the delayed group will continue their usual activities. This last group will receive baby HABIT-ILE intervention after the 3 months assessment. Primary outcome will be the Mini-Assisting Hand Assessment. Secondary outcomes will include behavioral assessments for gross and fine motricity, visual – cognitive – language abilities as well as magnetic resonance imaging and kinematics measures. Moreover, parents will determine and score child-relevant goals and fill out questionnaires of participation, daily activities and mobility.

Ethics and dissemination: Full ethical approval has been obtained by the *Comité d'éthique Hospitalo-Facultaire/Université catholique de Louvain*, Brussels (2013/01MAR/069 B403201316810g). The recommendations of the ethical board and the Belgian law of May 7th 2004 concerning human experiments will be followed. Parents will sign a written informed consent ahead of participation. Findings will be published in peer-reviewed journals and conference presentations.

Trial registration number: NCT04698395. Registered on the International Clinical Trials Registry Platform (ICTRP) on December 2, 2020 and NIH Clinical Trials Registry on January 6, 2021.

URL of trial registry record: https://clinicaltrials.gov/ct2/show/NCT04698395?term=bleyenheuft&draw=1&rank=7

For peer review only

Strengths and limitations of this study:

- This study is a randomized controlled trial assessing motor and non-motor changes after an intensive intervention of motor activities in infants with unilateral cerebral palsy (CP) aged 6 to 18 months (corrected if preterm).
- This study will be the first RCT to apply a bimanual and locomotor intervention in this age group and to assess neuroplastic changes after an intervention in infants with unilateral CP.
- The primary outcome (Mini-Assisting Hand Assessment) and the secondary motor outcome (Gross Motor Function Measure-66) will be videotaped and blindly scored.
- Recruiting 24 pairs of infants in a narrow age range who fit our inclusion criteria such as an early diagnosis of unilateral CP might be a challenge.

Keywords: cerebral palsy, infants, intensive rehabilitation, neuroplasticity, HABIT-ILE, randomized controlled trial.

Word count: 3731

For peer review only

For peer review only - http://bmjopen.bmj.com/site/about/guidelines.xhtml

Introduction

Cerebral palsy (CP), the most prevalent pediatric motor disability, is a group of disorders of movement and posture due to non-progressive brain damage in the developing brain occurring in 1 out of 500 neonates (1). Depending on the location of the brain damage, children can present motor impairments (2), as well as associated symptoms including sensory, cognitive and visuo-spatial impairments (1). These impairments generally lead to highly variable limitations both in gross motor function and manual abilities, which may greatly impair the future execution of many activities of daily living (ADL; such as dressing, eating, toileting ...), affecting both the lifelong autonomy and the quality of life of these children (3–5).

As the brain damage is non-evolutive, the main focus of management of children with CP is currently the treatment of the symptoms, most of the time through neurorehabilitation based on neurodevelopmental approaches, usually a few hours a week including a mix of stretching and movements manually guided towards "normality" (6). However, the efficiency of these approaches is debated (6,7). In contrast, there is strong evidence that more recent intensive interventions based on motor skill learning principles are more efficient in inducing functional and neuroplastic changes, across age groups and disability levels, compared to traditional non-intensive neurorehabilitation (7–13).

The intensive interventions are organized as blocks of training using motor learning concepts (practice specificity, context of learning, feedback, etc)(14), aiming to elicit practice-induced brain changes (neuroplasticity) arising from repetition, increasing movement complexity, motivation and reward (15,16). Though efficient to improve motor abilities, these interventions remain limited to the mere treatment of symptoms.

Research using animal models (17,18) suggests that intensive motor skill training in infants with CP may significantly reduce or even prevent maladaptive neuroplastic changes

For peer review only - http://bmjopen.bmj.com/site/about/guidelines.xhtml

consecutive to the brain lesion, potentially addressing some of the root causes of CP. More specifically, the initial brain damage induces a cascade of secondary neuroplastic damages that account for the motor disability: chronic inflammation (19–21), alterations in the organization of the corticospinal tract (CST) (22,23) and underdeveloped CST projections at the spinal level (24,25). Moreover, due to the perception-action loop, the resulting motor deficit may in turn affect the developmental course of other functions such as attentional, visual and spatial cognition (26).

Only a few motor intensive intervention trials have been carried out in young children under 3 years old (27). These studies have focused on children with unilateral CP and evaluated modified forms of Constraint-Induced Movement Therapy (CIMT) (28–32) and environmental enrichment intervention (33). These studies have provided moderate support in favor of CIMT and enrichment intervention. Higher daily intensity of training (30,31) thereby seems to induce larger effects than lower daily intensity training (28,29,32). Even though lower extremities are commonly affected in children with CP, CIMT focuses solely on the upper extremities (28–32). Moreover, none of these studies have investigated the possible impact of these interventions on organization and projections of the CST, as well as on non-motor functions.

Hand and Arm Bimanual Intensive Therapy Including Lower Extremities (HABIT-ILE), developed by Bleyenheuft and Gordon (2014), is an intensive intervention based on motor skill learning principles which focuses on a bimanual use associated to postural and/or gross motor stimulation (10). Its efficiency was demonstrated for both upper and lower extremities in children with unilateral (8,34) and bilateral CP (9) from 1 to 18 years old. An impact has been observed on motor function, visuospatial abilities and improvement of brain structure in children (35). Such an intervention has never been attempted in infants aged between 6 to 18 months with CP to tentatively prevent secondary neurological damages or developmental trajectories.

For peer review only - http://bmjopen.bmj.com/site/about/guidelines.xhtml

For peer review only

Aims and hypotheses

This study aims to demonstrate the effectiveness and feasibility of an intensive motor skill learning intervention (baby HABIT-ILE) in preventing secondary damage and in inducing changes in motor and non-motor functions in children with unilateral CP, compared to a control intervention (usual activities). We hypothesize that the baby HABIT-ILE intervention will induce greater improvements in the immediate group compared to usual activities of the delayed group. Moreover, possible differences related to therapy onset will be observed, with a potential disadvantage for the delayed group due to the influence of a three months-lag in receiving intensive intervention during their specific window of opportunity. A randomized controlled trial (RCT) was designed to verify our hypothesis after 2 weeks of treatment or control intervention, and at 3 months follow up. The results, scored by experts for the tests and by parents for the questionnaires, will be correlated with magnetic resonance imagery (MRI) changes.

Methods and analysis

Study design

This RCT will follow a two-parallel group design, involving forty-eight infants with unilateral CP between 6 and 18 months (corrected age). Assessments will be performed at the Institute of Neuroscience of the Université catholique de Louvain (UCLouvain) in Brussels, Belgium, at 3 time points: baseline (T0), 1 month after baseline (T1) and 3 months after baseline (T2). The between-group comparisons will be carried out between T0 and T1 and between T0 and T2. The delayed group will receive after the third assessment the baby HABIT-ILE intervention. A supplementary assessment 6 months after baseline (T3) will allow investigating the influence of the onset of therapy between both groups, and the persistence of the therapeutic effects at long term in the immediate group. At each assessment time, participants will follow motor and

For peer review only - http://bmjopen.bmj.com/site/about/guidelines.xhtml

non-motor-, functionals skills-, participation-, kinematics- and neuroimaging assessments (see Fig.1).

The CONSORT statement for non-pharmaceutical and pragmatic trials will be used for the structure of this study and the SPIRIT guidelines were used for the writing of this protocol. The study will be conducted from February 2021 to December 2023.

Recruitment, inclusion and exclusion criteria

Forty-eight infants will be included. They will be recruited from rehabilitation centers of Belgium. Spontaneous applications from parents will also be considered.

To be eligible for inclusion, infants must be:

- ✓ Aged (corrected age if preterm birth) 6 to 18 months at the first assessment;
- ✓ Diagnosed with unilateral CP or suspicion because of asymmetry or at risk of developing unilateral CP;
- ✓ Able to follow instructions and complete testing according to the age.

Infants will be excluded if they have:

- ☒ Uncontrolled seizures;
- ☒ Botulinum toxin injections, orthopedic surgery or specific intensive therapy within 6 months before and until the end of the study;
- ☒ Implants or other metallic devices that are contraindications to MRI;
- ☒ Severe visual or cognitive impairments preventing the child from being interested in simple games.

Sample size calculation

The sample size calculation was performed based on the Assisting Hand Assessment (AHA(36)) score of a HABIT-ILE pilot study performed in 10 infants with unilateral CP (34).

For peer review only - http://bmjopen.bmj.com/site/about/guidelines.xhtml

In this pilot study, toddlers with unilateral CP (1 to 4 years old) encountered an intensive intervention of 50 hours over 2 weeks, as in the present study. A mean improvement of 8,2 AHA units (standard deviation (SD)=5.9 AHA units) after the intensive therapy was reported. Thus, our hypothesis is an incremental improvement of 1 SD between the immediate group and the delayed group at second and third assessments (between group difference =7,2, SD=5,9). With α=0.05 and a 1-β=0.9, a sample size of 15 participants per group is required. Considering potential dropouts and data loss in MRI, 24 participants will be included in each group (48 in total).

Randomization process

A pair-matched randomization will be used. Group allocation to either immediate or delayed condition will be randomly determined within each pair using an online computer-based randomization sequence (37). Children will be matched regarding age (± 4 months) and etiology of CP (brain malformation / periventricular white matter lesion / grey matter lesion; following Krägeloh-Mann's classification (2)) before the baseline assessment and collection of informed consent. If possible, children will also be matched considering the affected hemisphere (right/left).

Blinding procedure

The participants and interventionists (therapists) will be blind to the group allocation. Study allocation codes will be used for analyses and all data will be anonymized. The Mini-AHA and the Gross Motor Function Measure-66 (GMFM-66) will be videotaped and blindly scored by external accredited/experienced raters unaware of group allocation or assessment time. Anonymized data will be stored in the Research Electronic Data Capture (REDCap) register which is hosted at the UCLouvain. Once the data collection is over, anonymized data will be analyzed.

For peer review only - http://bmjopen.bmj.com/site/about/guidelines.xhtml

Study Interventions

Control intervention: Usual activities

Usual activities have been chosen as control intervention since spontaneous activity of infants is oriented towards the discovery of their environment with motor activities in a nursery reaching around 5 hours per day (38).The infants allocated to the delayed group will continue, between the first and the second assessments, their usual activities including nursery and ongoing therapy, generally a few hours of neurodevelopmental therapy per week. Wrist movement sensors will record the amount of motor activity during 5 days, 5 hours a day, to document the percentage of total time spent in movement during the control intervention.

Treatment intervention: baby HABIT-ILE

The intensive motor skill training consists of an adaptation of HABIT-ILE (8–10). This intervention, which was developed initially for children with CP over 6 years old, is carried out in a camp setting, with structured tasks of increasing motor difficulty and functional activities that require the use of both hands whilst sustaining postural activity or activities of the lower extremities. The therapy, using games and functional activities, is delivered in a child-friendly environment. The tasks are chosen according to individualized functional goals previously defined by the parents (e.g. drinking or sitting autonomously, turning the pages of a book, moving around). Although the basic principles of HABIT-ILE will be followed (10), the toys, tasks and activities will be adapted to the age of the infants. The duration of activities as well as lower extremities/postural requests will also be adapted to each infant depending on his/her functional goals and individual characteristics (motor and attentional level, for instance). Twelve infants will undergo rehabilitation at the same time during one intensive camp of 2 weeks (4 camps in total); each infant will have a full-time therapist dedicated to his/her

For peer review only - http://bmjopen.bmj.com/site/about/guidelines.xhtml

treatment. The monitoring of the guidelines and the fidelity to the treatment principles will be ensured throughout the whole intervention by experienced HABIT-ILE supervisors. As 50 to 60 hours of intensive therapy are needed to induce long-term motor changes (39,40), this project, in agreement with the usual motor activity time (38) and the need for infants to rest, will include 5 hours per day of therapy with the same therapist for a total of 50 hours (5days/week). HABIT-ILE for infants has been tested in a pilot study which has confirmed previous evidence that this therapy intensity is both practically feasible and efficient for children at that age (34). Infants will follow the intervention during 3 hours in the morning (with a short break for those who need a morning nap), they will subsequently have 2 hours of nap-break around noon, and will complete 2 additional hours of intervention in the afternoon.

Data collection and data management

An "assessment log sheet" including patient code, date of assessment, test procedure, mood of the child and context during the data acquisition will be used at each assessment. This information will be collected on REDCap (41,42) register. All children (immediate and delayed groups) will be seen at the 4 assessments times. At each assessment time, children will complete all the assessments listed below, except the ophthalmological assessment which is only carried out once.

Each assessment time will be performed during one day. To avoid tiredness and ensure quality testing, 2 blocks of maximum 1h30 of functional assessment will be spread over the day. Rest periods will be planned between blocks and parents will be present during the whole day.

Outcomes

Outcomes of the study were chosen to cover the 3 levels of the International Classification of Functioning, Disability and Health: Children & Youth version (ICF-CY)(43). A daily logbook filled by the interventionists during the whole therapy time will be used to examine the

For peer review only - http://bmjopen.bmj.com/site/about/guidelines.xhtml

feasibility of the intervention. Interventionists will write the time and the type of activities for each participant during the whole camp duration.

Primary outcome

The Mini-AHA, as primary outcome, measures the use of the more affected hand collaboratively with the less affected hand in bimanual activities. This videotaped test targets the activity domain of the ICF-CY and uses Rasch model analysis. The score is expressed on a logit based AHA-unit scale ranging from 0 to 100 (the higher the score, the better the ability). This assessment is responsive, reliable, and valid for use in children with unilateral CP (44).

Secondary outcomes

These outcomes concern the three levels of the ICF-CY. First, the body functions and structures will be studied through ophthalmological and neuroimaging assessments. Second, the activity level of the children will be assessed with the GMFM-66 (45), the Bayley Scales of Infant and toddler Development-Third edition (BSID-III) (46), the kinematics assessment (Vicon 3D motion system (47)), the kinetics assessment (Xsens-dot movement sensors (48)) and the visuospatial battery "Batterie d'évaluation du Jeune Enfant" (BAJE) (49). Moreover, questionnaires including the Pediatric Evaluation of Disability Inventory - Computer Adaptive Test (PEDI-CAT) (50,51) and the Canadian Occupational Performance Measure (COPM) (52) will assess both activity and participation levels. Third, the Young Children's Participation and Environment Measure (YC-PEM) (53), for its part, will only assess the participation level.

Body functions and structures assessments

To determine neuroplastic changes, MRI datasets will be acquired. Those assessments will take place in the Saint-Luc University Hospital in Brussels using a 3 Tesla MRI (SIGNA™ Premier, General Electric), equipped with a 48-channel head coil. The MRI procedure will consist in night scanning during natural, non-sedated sleep. Infants will undergo a standardized, gradual,

For peer review only - http://bmjopen.bmj.com/site/about/guidelines.xhtml

adaptation home program to accustom them to the specific conditions of the MRI environment (wearing earplugs and careful examination of each infant's sleep routines (54)). Adapted transfer material allowing the infant to fall asleep in a child-friendly environment in the MRI service and to be transferred asleep in the machine will be used. The total time of MRI is estimated to 35 minutes. MRI changes will be measured with anatomical imaging (T1, T2 and multi-shell diffusion sequences) and functional imaging (resting-state sequence). More precisely, our analyses will include the classical diffusion tensor imaging (DTI) indicators of fractional anisotropy, radial diffusivity, axial diffusivity, and mean diffusivity (55,56) as well as cortical thickness. In addition, new advanced model-based microstructural features based on angular weighting will be used to investigate microscopic level of training-induced changes (57,58). At macroscopic level, tractography will be used to highlight potential changes in CST and optic radiation organization (59,60).

All infants will encounter an ophthalmological assessment at the last assessment (T3) by ophthalmologists from the Saint-Luc University Hospital, Brussels. A standard battery of ophthalmological tests for infants will be used (Hirshberg(61), Gracis biprism (62), Lang II(63,64), Cover Test (65),ocular motility, Cardiff test (66), empty refraction, refraction under cycloplegia, dilated fundus, etc). This assessment will allow to exclude visual impairments of peripheral origin, to document visual impairments and to describe the baseline of the ophthalmological condition of these infants.

Activity assessments

Gross motor function will be measured using the GMFM-66 (45). This tool is a clinical assessment for children with CP allowing the measure of changes in gross motor function over time or following an intervention. The items cover a large spectrum of activities divided into 5 domains: 'lying and rolling', 'sitting', 'crawling and kneeling', 'standing', and 'walking, running and jumping'. This test will be videotaped and uses Rasch model analysis. The score

For peer review only - http://bmjopen.bmj.com/site/about/guidelines.xhtml

is expressed on percentage of logits. This test is responsive (67), reliable and valid for infants with CP (68).

The BSID-III (46) will be used to assess cognitive, motor and language development. The cognitive scale includes attention and habituation tasks, problem solving, play tasks, object assembly, concept grouping and memory tasks. The motor scale, divided into two subscales includes assessments of gross and fine motor skills such as grasping, sitting, stacking blocks and climbing stairs. Finally, the language scale, also divided into two subscales (receptive and expressive communication) includes understanding and expression of language (recognition of objects and people, following directions, naming and recognizing objects and pictures, etc). Raw scores of the items are converted to scaled scores and composite scores (mean 100, SD 15) ranging from 40 to 160 (higher scores indicate better performance). This assessment has been shown to be reliable and valid in young children with CP (69).

Kinematics assessments will be used to determine qualitative changes in upper extremities movement characteristics. Markers will be positioned on the head (using a headband), acromions, elbows and wrists of the child. Children will sit on an adapted chair, in front of a table. If the child does not sit up independently, an adult will support his/ her trunk. The first task will consist of a unimanual reaching of a toy, with the less-affected hand and the more-affected hand, separately. The second task will be bimanual: children will be asked to tap 2 cymbals against each other. Through a 3D motion system (Vicon Motion Systems, Oxford, UK (47)) the straightness (percentage of upper extremities trajectory during a reaching task) and smoothness (variability of movement during a reaching task) of the upper extremities will be studied for each task. Also, the time of activity (time from onset to end of the task in seconds) during a reaching task will be analysed.

Quantitative changes of physical activity will also be measured with two movement sensors (Xsens-Dot (48)), placed on each wrist during 5 hours a day for 5 days at home and during the

For peer review only - http://bmjopen.bmj.com/site/about/guidelines.xhtml

camp duration (documented by a logbook). The percentage of total time spent in movement (i.e. crawling, walking and running) will be measured and calculated in terms of the changes in the acceleration (m/s^2).

In addition, the visuospatial battery BAJE (49) will be used to assess changes in visuo-spatial attention and visuo-attentional abilities (70). The BAJE is an adaptation of the Evaluation of Visuo-spatial Abilities (EVA) (70–72) which is designed to test visuospatial and visuo-attentional abilities of children from 3 months of age, developed and validated by Sylvie Chokron's team. This normalized and standardized battery includes simple tasks such as photomotor reflex and blink reflex at threat, visual fixation, light detection, visual pursuit, visual filed/close visual field, visuo-motor coordination, … In this study, through three different tasks of this battery (visuo-motor coordination, visual field and eye pursuit), we will evaluate the presence and severity of the most common cerebral visual impairments (visual field defect, visuo-attentional, visuospatial and visuo-motor deficits).

The 'daily activities' and 'mobility' domains of the PEDI-CAT will be used to evaluate the capacity of upper and lower extremities of children with CP during specific activities (50). The scores are computed through the PEDI-CAT software (scores reported in scaled scores). This questionnaire is sensitive for children with CP (73).

The COPM (52), a semi-structured interview performed by a trained examiner, will be used to define the therapeutic goals and to quantify the child's performance (1 to 10 scale) in relation to these goals, as well as the satisfaction (1 to 10 scale) of the parents regarding their child's achievement. Total score is the average of the scores for performance and satisfaction separately.

Participation assessments

For peer review only - http://bmjopen.bmj.com/site/about/guidelines.xhtml

Participation will be evaluated using the PEDI-CAT(74), the COPM (52) and the YC-PEM (53). Based on different children's activities, the YC-PEM is a parents-filled questionnaire evaluating the level of participation and the quality of the environment across three settings: home, daycare/preschool and community. For each type of activity, caregivers assess multiple dimensions of the child's participation: frequency, level of involvement, caregiver's percent desire for change and perceived impact of environmental support. It has a good intra-rater and inter-rater reliability (53).

Statistical analysis

A two-way repeated measures analysis of variance (RM ANOVA) (2 groups [immediate vs delayed] x 3 assessment times [T0, T1, T2]) with repeated measures on the time factor and Holm– Šidák post hoc analyses for pairwise multiple comparisons (or non-parametric equivalent when normality and homoscedasticity are not met) will be used for the primary outcome, the Mini-AHA, and the secondary outcomes. Significant differences will be statistically set at $\alpha = 0.05$.

Ethics and dissemination

Full ethical approval has been obtained for this study protocol by the Comité d’éthique Hospitalo-Facultaire/UCLouvain, Brussels (reference number: 2013/01MAR/069 B403201316810g). An information letter will be provided to the parents. They will be asked to sign a consent form if they comply to their child’s participation. The parents will be informed during the recruitment process of their right to withdraw their child at any time without prejudice. All data collected will be anonymous and will be stored by the UCLouvain. Findings from this study will be published in peer-reviewed scientific journals and conference presentations. This study has been registered on NIH Clinical Trials Registry on January, 6 2021(NCT04698395

For peer review only

For peer review only - http://bmjopen.bmj.com/site/about/guidelines.xhtml

https://clinicaltrials.gov/ct2/show/NCT04698395?term=bleyenheuft&draw=1&rank=7). After contacting the corresponding author and signing a data sharing agreement, a digital object identifier could be shared to access selected data.

Patient and public involvement

Patients and the public were not involved in the design, or conduct, or reporting, or dissemination plans of this research.

Conclusion

This RCT has been designed to substantiate evidence of the efficacy of the baby HABIT-ILE intervention to provide functional and neuroplastic changes in infants with unilateral CP. By implementing the baby HABIT-ILE intervention very early in the child's development during a specific window of opportunity, we hope to prevent the onset of secondary damages and, consequently, to potentiate the long-term motor abilities of the children and increase their autonomy.

Abbreviations

AHA: Assisting Hand Assessment; RM-ANOVA: Repeated Measures Analysis of Variance; BAJE: Batterie d'évaluation du jeune enfant ; BSID-III: Bayley Scale of Infant and toddler Development-Third edition; COPM: Canadian Occupational Performance Measure; CP: Cerebral Palsy; CST: Corticospinal Tract; DTI: Diffusion Tensor Imaging; GMFM-66: Gross Motor Function Measure-66; HABIT-ILE: Hand and Arm Bimanual Intensive Therapy Including Lower Extremities; MRI: Magnetic Resonance Imaging; PEDI-CAT: Pediatric Evaluation of Disability Inventory - Computer Adaptive Test; RCT: Randomized Controlled

For peer review only - http://bmjopen.bmj.com/site/about/guidelines.xhtml

Trial; REDCap: Research Electronic Data Capture; UCLouvain: Université catholique de Louvain; YC-PEM: Young Children's Participation and Environment Measure

Author contributions

YB, LD, BM, YV contributed to study design and obtained funding for the research study. AC and EH first drafted this protocol. YB, RA, DEK, EH, AC and EG will be involved in the implementation of the therapy. EH, AC, EG, DEK and RA will recruit the participants and perform the assessments. YB, AC, EH, LD, DEK and RA will contribute to data analysis. All authors contributed to the writing of this manuscript and have critically reviewed and approved the final version.

Funding

This study is part of a larger study funded by the Fédération Wallonie-Bruxelles through the program "*Action de Recherche Concertée"* of the UCLouvain (20/25-105), Belgium. Partial grant specifically dedicated to the visual part has also been provided by the "*Fondation JED-Belgique*". EH has a grant from the "*Fonds de la Recherche Scientifique - FNRS"*(A1.21302.026-F). The funding bodies have reviewed this protocol during the selection process, but after this initial point, have participated neither in the study design nor in data collection, analysis, interpretation data, nor in writing the manuscript.

Competing interests

The authors have no conflict of interest to disclose.

For peer review only - http://bmjopen.bmj.com/site/about/guidelines.xhtml

REFERENCES

1. Graham HK, Rosenbaum P, Paneth N, Dan B, Lin JP, Damiano DL, et al. Cerebral palsy. Nat Rev Dis Primers [Internet]. 2016/05/18. 2016;2:15082. Available from: https://www.nature.com/articles/nrdp201582

2. Krägeloh-Mann I, Horber V. The role of magnetic resonance imaging in elucidating the pathogenesis of cerebral palsy: a systematic review. Dev Med Child Neurol [Internet]. 2007/01/27. 2007;49(2):144–51. Available from: https://onlinelibrary.wiley.com/doi/10.1111/j.1469-8749.2007.00144.x

3. Colver A, Rapp M, Eisemann N, Ehlinger V, Thyen U, Dickinson HO, et al. Self-reported quality of life of adolescents with cerebral palsy: a cross-sectional and longitudinal analysis. Lancet [Internet]. 2015 Feb 21 [cited 2022 Nov 15];385(9969):705–16. Available from: https://pubmed.ncbi.nlm.nih.gov/25301503/

4. Dickinson HO, Parkinson KN, Ravens-Sieberer U, Schirripa G, Thyen U, Arnaud C, et al. Self-reported quality of life of 8-12-year-old children with cerebral palsy: a cross-sectional European study. Lancet [Internet]. 2007 Jun 30 [cited 2022 Nov 15];369(9580):2171–8. Available from: https://pubmed.ncbi.nlm.nih.gov/17604799/

5. Kerr C, Mcdowell B, Mcdonough S. The relationship between gross motor function and participation restriction in children with cerebral palsy: an exploratory analysis. Child Care Health Dev [Internet]. 2007 Jan [cited 2022 Nov 15];33(1):22–7. Available from: https://pubmed.ncbi.nlm.nih.gov/17181749/

6. Te Velde A, Morgan C, Finch-Edmondson M, McNamara L, McNamara M, Paton MCB, et al. Neurodevelopmental Therapy for Cerebral Palsy: A Meta-analysis. Pediatrics [Internet]. 2022 Jun 1 [cited 2023 Apr 18];149(6). Available from: https://pubmed.ncbi.nlm.nih.gov/35607928/

7. Novak I, Morgan C, Fahey M, Finch-Edmondson M, Galea C, Hines A, et al. State of the Evidence Traffic Lights 2019: Systematic Review of Interventions for Preventing and Treating Children with Cerebral Palsy. Curr Neurol Neurosci Rep [Internet]. 2020/02/23. 2020;20(2):3. Available from: https://www.ncbi.nlm.nih.gov/pmc/articles/PMC7035308/pdf/11910_2020_Article_1022.pdf

8. Bleyenheuft Y, Arnould C, Brandao MB, Bleyenheuft C, Gordon AM. Hand and Arm Bimanual Intensive Therapy Including Lower Extremity (HABIT-ILE) in Children With Unilateral Spastic Cerebral Palsy: A Randomized Trial. Neurorehabil Neural Repair [Internet]. 2014/12/21. 2015;29(7):645–57. Available from: https://journals.sagepub.com/doi/pdf/10.1177/1545968314562109

9. Bleyenheuft Y, Ebner-Karestinos D, Surana B, Paradis J, Sidiropoulos A, Renders A, et al. Intensive upper- and lower-extremity training for children with bilateral cerebral palsy: a quasi-randomized trial. Dev Med Child Neurol [Internet]. 2017 Jun 1 [cited 2022 Nov 3];59(6):625–33. Available from: https://pubmed.ncbi.nlm.nih.gov/28133725/

10. Bleyenheuft Y, Gordon AM. Hand-arm bimanual intensive therapy including lower extremities (HABIT-ILE) for children with cerebral palsy. Phys Occup Ther Pediatr [Internet]. 2014/10/02. 2014;34(4):390–403. Available from: https://www.tandfonline.com/doi/full/10.3109/01942638.2014.932884

For peer review only - http://bmjopen.bmj.com/site/about/guidelines.xhtml

11. Hatem SM, Saussez G, della Faille M, Prist V, Zhang X, Dispa D, et al. Rehabilitation of Motor Function after Stroke: A Multiple Systematic Review Focused on Techniques to Stimulate Upper Extremity Recovery. Front Hum Neurosci [Internet]. 2016 Sep 13 [cited 2022 Nov 17];10(SEP2016):442. Available from: http://www.ncbi.nlm.nih.gov/pubmed/27679565

12. Brandão MB, Mancini MC, Ferre CL, Figueiredo PRP, Oliveira RHS, Gonçalves SC, et al. Does Dosage Matter? A Pilot Study of Hand-Arm Bimanual Intensive Training (HABIT) Dose and Dosing Schedule in Children with Unilateral Cerebral Palsy. Phys Occup Ther Pediatr [Internet]. 2018 May 27 [cited 2023 Apr 18];38(3):227–42. Available from: https://pubmed.ncbi.nlm.nih.gov/29240518/

13. Hoare BJ, Wallen MA, Thorley MN, Jackman ML, Carey LM, Imms C. Constraint-induced movement therapy in children with unilateral cerebral palsy. Cochrane Database Syst Rev [Internet]. 2019 Apr 1 [cited 2023 Apr 18];4(4). Available from: https://pubmed.ncbi.nlm.nih.gov/30932166/

14. Schmidt RA, Lee TD, Winstein C, Wulf G, Zelaznik HN. Motor control and learning: A behavioral emphasis. Human kinetics; 2018.

15. Nudo RJ. Adaptive plasticity in motor cortex: implications for rehabilitation after brain injury. J Rehabil Med [Internet]. 2003 [cited 2023 Apr 18];(41 Suppl):7–10. Available from: https://pubmed.ncbi.nlm.nih.gov/12817650/

16. Kleim JA, Hogg TM, VandenBerg PM, Cooper NR, Bruneau R, Remple M. Cortical synaptogenesis and motor map reorganization occur during late, but not early, phase of motor skill learning. J Neurosci [Internet]. 2004 Jan 21 [cited 2023 Apr 18];24(3):628–33. Available from: https://pubmed.ncbi.nlm.nih.gov/14736848/

17. Friel KM, Chakrabarty S, Martin JH. Pathophysiological mechanisms of impaired limb use and repair strategies for motor systems after unilateral injury of the developing brain. Dev Med Child Neurol [Internet]. 2013 Nov [cited 2023 Apr 13];55 Suppl 4(SUPPL.4):27–31. Available from: https://pubmed.ncbi.nlm.nih.gov/24237276/

18. Friel KM, Drew T, Martin JH. Differential activity-dependent development of corticospinal control of movement and final limb position during visually guided locomotion. J Neurophysiol [Internet]. 2007 May [cited 2023 Apr 13];97(5):3396–406. Available from: https://pubmed.ncbi.nlm.nih.gov/17376849/

19. Baud O, Daire JL, Dalmaz Y, Fontaine RH, Krueger RC, Sebag G, et al. Gestational hypoxia induces white matter damage in neonatal rats: a new model of periventricular leukomalacia. Brain Pathol [Internet]. 2004/03/05. 2004;14(1):1–10. Available from: https://onlinelibrary.wiley.com/doi/abs/10.1111/j.1750-3639.2004.tb00492.x?sid=nlm%3Apubmed

20. Favrais G, van de Looij Y, Fleiss B, Ramanantsoa N, Bonnin P, Stoltenburg-Didinger G, et al. Systemic inflammation disrupts the developmental program of white matter. Ann Neurol [Internet]. 2011/07/29. 2011;70(4):550–65. Available from: https://onlinelibrary.wiley.com/doi/abs/10.1002/ana.22489

21. van Steenwinckel J, Schang AL, Sigaut S, Chhor V, Degos V, Hagberg H, et al. Brain damage of the preterm infant: new insights into the role of inflammation. Biochem Soc Trans [Internet]. 2014/03/22. 2014;42(2):557–63. Available from:

For peer review only - http://bmjopen.bmj.com/site/about/guidelines.xhtml

https://portlandpress.com/biochemsoctrans/article-abstract/42/2/557/66817/Brain-damage-of-the-preterm-infant-new-insights?redirectedFrom=fulltext

22. Eyre JA, Taylor JP, Villagra F, Smith M, Miller S. Evidence of activity-dependent withdrawal of corticospinal projections during human development. Neurology [Internet]. 2001 Nov 13 [cited 2022 Nov 3];57(9):1543–54. Available from: https://pubmed.ncbi.nlm.nih.gov/11706088/

23. Staudt M. Brain Plasticity Following Early Life Brain Injury: Insights From Neuroimaging. Vol. 34, Seminars in Perinatology. 2010. p. 87–92.

24. Martin JH, Lee SJ. Activity-dependent competition between developing corticospinal terminations. Neuroreport. 1999/08/10. 1999;10(11):2277–82.

25. Martin JH. The corticospinal system: from development to motor control. Neuroscientist [Internet]. 2005/03/05. 2005;11(2):161–73. Available from: https://journals.sagepub.com/doi/10.1177/1073858404270843?url_ver=Z39.88-2003&rfr_id=ori:rid:crossref.org&rfr_dat=cr_pub%3Dpubmed

26. Chokron S, Dutton GN. Impact of Cerebral Visual Impairments on Motor Skills: Implications for Developmental Coordination Disorders. Front Psychol [Internet]. 2016 Oct 4 [cited 2022 Nov 3];7(OCT). Available from: https://pubmed.ncbi.nlm.nih.gov/27757087/

27. Damiano DL, Longo E. Early intervention evidence for infants with or at risk for cerebral palsy: an overview of systematic reviews. Dev Med Child Neurol [Internet]. 2021/04/08. 2021; Available from: https://onlinelibrary.wiley.com/doi/10.1111/dmcn.14855

28. Eliasson AC, Nordstrand L, Ek L, Lennartsson F, Sjöstrand L, Tedroff K, et al. The effectiveness of Baby-CIMT in infants younger than 12 months with clinical signs of unilateral-cerebral palsy; an explorative study with randomized design. Res Dev Disabil. 2017/11/28. 2018;72:191–201.

29. Eliasson AC, Shaw K, Berg E, Krumlinde-Sundholm L. An ecological approach of Constraint Induced Movement Therapy for 2-3-year-old children: a randomized control trial. Res Dev Disabil [Internet]. 2011 Nov [cited 2022 Nov 3];32(6):2820–8. Available from: https://pubmed.ncbi.nlm.nih.gov/21700416/

30. Taub E, Ramey SL, DeLuca S, Echols K. Efficacy of constraint-induced movement therapy for children with cerebral palsy with asymmetric motor impairment. Pediatrics [Internet]. 2004 Feb [cited 2022 Nov 3];113(2):305–12. Available from: https://pubmed.ncbi.nlm.nih.gov/14754942/

31. Deluca SC, Case-Smith J, Stevenson R, Ramey SL. Constraint-induced movement therapy (CIMT) for young children with cerebral palsy: effects of therapeutic dosage. J Pediatr Rehabil Med [Internet]. 2012 [cited 2022 Nov 3];5(2):133–42. Available from: https://pubmed.ncbi.nlm.nih.gov/22699104/

32. Nordstrand L, Holmefur M, Kits A, Eliasson AC. Improvements in bimanual hand function after baby-CIMT in two-year old children with unilateral cerebral palsy: A retrospective study. Res Dev Disabil. 2015/06/24. 2015;41–42:86–93.

For peer review only

33. Morgan C, Novak I, Dale RC, Guzzetta A, Badawi N. Single blind randomised controlled trial of GAME (Goals - Activity - Motor Enrichment) in infants at high risk of cerebral palsy. Res Dev Disabil. 2016/05/11. 2016;55:256–67.

34. Araneda R, Klöcker A, Ebner-Karestinos D, Sogbossi ES, Renders A, Saussez G, et al. Feasibility and effectiveness of HABIT-ILE in children aged 1 to 4 years with cerebral palsy: A pilot study. Ann Phys Rehabil Med. 2021;64(3):3–6.

35. Bleyenheuft Y, Dricot L, Ebner-Karestinos D, Paradis J, Saussez G, Renders A, et al. Motor Skill Training May Restore Impaired Corticospinal Tract Fibers in Children With Cerebral Palsy. Neurorehabil Neural Repair [Internet]. 2020 Jun 1 [cited 2022 Nov 3];34(6):533–46. Available from: https://pubmed.ncbi.nlm.nih.gov/32407247/

36. Krumlinde-Sundholm L, Eliasson AC. Development of the Assisting Hand Assessment: a Rasch-built measure intended for children with unilateral upper limb impairments. Scand J Occup Ther. 2003;10(1):16–26.

37. Research Randomizer [Internet]. Available from: https://randomizer.org/

38. Van Cauwenberghe E, Gubbels J, De Bourdeaudhuij I, Cardon G. Feasibility and validity of accelerometer measurements to assess physical activity in toddlers. Int J Behav Nutr Phys Act [Internet]. 2011 Jun 26 [cited 2023 Jan 5];8. Available from: https://pubmed.ncbi.nlm.nih.gov/21703004/

39. Brandão MB, Mancini MC, Ferre CL, Figueiredo PRP, Oliveira RHS, Gonçalves SC, et al. Does Dosage Matter? A Pilot Study of Hand-Arm Bimanual Intensive Training (HABIT) Dose and Dosing Schedule in Children with Unilateral Cerebral Palsy. Phys Occup Ther Pediatr [Internet]. 2018 May 27 [cited 2022 Nov 8];38(3):227–42. Available from: https://pubmed.ncbi.nlm.nih.gov/29240518/

40. Sakzewski L, Provan K, Ziviani J, Boyd RN. Comparison of dosage of intensive upper limb therapy for children with unilateral cerebral palsy: how big should the therapy pill be? Res Dev Disabil [Internet]. 2015 Feb 1 [cited 2022 Nov 8];37:9–16. Available from: https://pubmed.ncbi.nlm.nih.gov/25460215/

41. Harris PA, Taylor R, Minor BL, Elliott V, Fernandez M, O'Neal L, et al. The REDCap consortium: Building an international community of software platform partners. J Biomed Inform [Internet]. 2019 Jul 1 [cited 2022 Nov 9];95. Available from: https://pubmed.ncbi.nlm.nih.gov/31078660/

42. Harris PA, Taylor R, Thielke R, Payne J, Gonzalez N, Conde JG. Research electronic data capture (REDCap)--a metadata-driven methodology and workflow process for providing translational research informatics support. J Biomed Inform [Internet]. 2009 Apr [cited 2022 Nov 9];42(2):377–81. Available from: https://pubmed.ncbi.nlm.nih.gov/18929686/

43. Organization WH. International Classification of Functioning, Disability, and Health: Children & Youth Version: ICF-CY. World Health Organization; 2007.

44. Greaves S, Imms C, Dodd K, Krumlinde-Sundholm L. Development of the Mini-Assisting Hand Assessment: evidence for content and internal scale validity. Dev Med Child Neurol [Internet]. 2013/08/01. 2013;55(11):1030–7. Available from: https://onlinelibrary.wiley.com/doi/pdfdirect/10.1111/dmcn.12212?download=true

For peer review only

45. Russell DJ, Avery LM, Walter SD, Hanna SE, Bartlett DJ, Rosenbaum PL, et al. Development and validation of item sets to improve efficiency of administration of the 66-item Gross Motor Function Measure in children with cerebral palsy. Dev Med Child Neurol [Internet]. 2009/10/09. 2010;52(2):e48-54. Available from: https://onlinelibrary.wiley.com/doi/10.1111/j.1469-8749.2009.03481.x

46. Bayley N. Bayley-III: Bayley Scales of infant and toddler development. Giunti OS; 2009.

47. Merriaux P, Dupuis Y, Boutteau R, Vasseur P, Savatier X. A study of vicon system positioning performance. Sensors. 2017;17(7):1591.

48. Ribera D'alcala' E, Voerman JA, Konrath JM, Vydhyanathan A. Xsens DOT Wearable Sensor Platform White Paper. 2021;

49. Petkovic M, Chokron S, Fagard J. Visuo-manual coordination in preterm infants without neurological impairments. Res Dev Disabil [Internet]. 2016 Apr 1 [cited 2022 Nov 9];51–52:76–88. Available from: https://pubmed.ncbi.nlm.nih.gov/26812594/

50. Kramer JM, Liljenquist K, Coster WJ. Validity, reliability, and usability of the Pediatric Evaluation of Disability Inventory-Computer Adaptive Test for autism spectrum disorders. Dev Med Child Neurol [Internet]. 2015/06/25. 2016;58(3):255–61. Available from: https://onlinelibrary.wiley.com/doi/pdfdirect/10.1111/dmcn.12837?download=true

51. Thompson S V., Cech DJ, Cahill SM, Krzak JJ. Linking the Pediatric Evaluation of Disability Inventory-Computer Adaptive Test (PEDI-CAT) to the International Classification of Function. Pediatr Phys Ther [Internet]. 2018 Apr 1 [cited 2023 Apr 18];30(2):113–8. Available from: https://pubmed.ncbi.nlm.nih.gov/29498960/

52. Dedding C, Cardol M, Eyssen IC, Dekker J, Beelen A. Validity of the Canadian Occupational Performance Measure: a client-centred outcome measurement. Clin Rehabil. 2004/10/12. 2004;18(6):660–7.

53. Khetani MA, Graham JE, Davies PL, Law MC, Simeonsson RJ. Psychometric properties of the Young Children's Participation and Environment Measure. Arch Phys Med Rehabil. 2014/12/03. 2015;96(2):307–16.

54. Dean DC, Dirks H, O'Muircheartaigh J, Walker L, Jerskey BA, Lehman K, et al. Pediatric neuroimaging using magnetic resonance imaging during non-sedated sleep. Pediatr Radiol [Internet]. 2014 Jan [cited 2022 Nov 9];44(1):64–72. Available from: https://pubmed.ncbi.nlm.nih.gov/23917588/

55. Beaulieu C. The basis of anisotropic water diffusion in the nervous system - a technical review. NMR Biomed [Internet]. 2002 Nov [cited 2023 Apr 13];15(7–8):435–55. Available from: https://pubmed.ncbi.nlm.nih.gov/12489094/

56. Alexander AL, Lee JE, Lazar M, Field AS. Diffusion tensor imaging of the brain. Neurotherapeutics [Internet]. 2007 Jul [cited 2023 Apr 13];4(3):316–29. Available from: https://pubmed.ncbi.nlm.nih.gov/17599699/

57. Rensonnet G, Scherrer B, Girard G, Jankovski A, Warfield SK, Macq B, et al. Towards microstructure fingerprinting: Estimation of tissue properties from a dictionary of Monte Carlo diffusion MRI simulations. Neuroimage [Internet]. 2019 Jan 1 [cited 2023 Apr 13];184:964–80. Available from: https://pubmed.ncbi.nlm.nih.gov/30282007/

For peer review only

For peer review only - http://bmjopen.bmj.com/site/about/guidelines.xhtml

58. Scherrer B, Schwartzman A, Taquet M, Sahin M, Prabhu SP, Warfield SK. Characterizing brain tissue by assessment of the distribution of anisotropic microstructural environments in diffusion-compartment imaging (DIAMOND). Magn Reson Med [Internet]. 2016 Sep 1 [cited 2023 Apr 13];76(3):963–77. Available from: https://pubmed.ncbi.nlm.nih.gov/26362832/

59. Baek SO, Jang SH, Lee E, Kim S, Hah JO, Park YH, et al. CST recovery in pediatric hemiplegic patients: Diffusion tensor tractography study. Neurosci Lett [Internet]. 2013 Dec 17 [cited 2023 Apr 13];557 Pt B(PB):79–83. Available from: https://pubmed.ncbi.nlm.nih.gov/24176879/

60. Araneda R, Ebner-Karestinos D, Dricot L, Herman E, Hatem SM, Friel KM, et al. Impact of early brain lesions on the optic radiations in children with cerebral palsy. Front Neurosci [Internet]. 2022 Oct 5 [cited 2023 Apr 13];16. Available from: https://pubmed.ncbi.nlm.nih.gov/36278011/

61. Jones R, Eskridge JB. THE HIRSCHBERG TEST — A RE-EVALUATION*. Optometry and Vision Science [Internet]. 1970;47(2). Available from: https://journals.lww.com/optvissci/Fulltext/1970/02000/THE_HIRSCHBERG_TEST___A_RE_EVALUATION_.4.aspx

62. Paris V. [Screening and treatment of fixation dominance in primary microstrabismus]. Bull Soc Belge Ophtalmol [Internet]. 1992;243:45–53. Available from: http://europepmc.org/abstract/MED/1302153

63. Joseph L. A New Stereotest. Journal of Pediatric Ophthalmology & Strabismus [Internet]. 1983 Mar 1;20(2):72–4. Available from: https://doi.org/10.3928/0191-3913-19830301-08

64. Ohlsson J, Villarreal G, Sjöström A, Abrahamsson M, Sjöstrand J. Screening for amblyopia and strabismus with the Lang II stereo card. Acta Ophthalmol Scand [Internet]. 2002 Apr 1;80(2):163–6. Available from: https://doi.org/10.1034/j.1600-0420.2002.800208.x

65. PELI ELI, McCORMACK G. Dynamics of cover test eye movements. Optometry and Vision Science. 1983;60(8):712–24.

66. Adoh TO, Woodhouse JM. The Cardiff acuity test used for measuring visual acuity development in toddlers. Vision Res. 1994;34(4):555–60.

67. Vos-Vromans DC, Ketelaar M, Gorter JW. Responsiveness of evaluative measures for children with cerebral palsy: the Gross Motor Function Measure and the Pediatric Evaluation of Disability Inventory. Disabil Rehabil [Internet]. 2005/11/22. 2005;27(20):1245–52. Available from: https://www.tandfonline.com/doi/abs/10.1080/09638280500076178

68. Wei S, Su-Juan W, Yuan-Gui L, Hong Y, Xiu-Juan X, Xiao-Mei S. Reliability and Validity of the GMFM-66 in 0- to 3-Year-Old Children with Cerebral Palsy. Am J Phys Med Rehabil [Internet]. 2006/01/24. 2006 Feb;85(2):141–7. Available from: http://journals.lww.com/00002060-200602000-00006

69. Gill K, Osiovich A, Synnes A, J AA, Grunau RE, Miller SP, et al. Concurrent Validity of the Bayley-III and the Peabody Developmental Motor Scales-2 at 18 Months. Phys Occup Ther Pediatr [Internet]. 2018/12/14. 2019;39(5):514–24. Available from: https://www.tandfonline.com/doi/full/10.1080/01942638.2018.1546255

For peer review only

For peer review only - http://bmjopen.bmj.com/site/about/guidelines.xhtml

70. Chokron S. Evaluation of visuo-spatial abilities (EVA): a simple and rapid battery to screen for CVI in young children. Impairment of vision due to disorders of the visual brain in childhood: a practical approach. 2015;

71. Cavézian C, Vilayphonh M, Vasseur V, Caputo G, Laloum L, Chokron S. Ophthalmic disorder may affect visuo-attentional performance in childhood. Child Neuropsychology. 2013 May 1;19(3):292–312.

72. Cavézian C, Vilayphonh M, de Agostini M, Vasseur V, Watier L, Kazandjian S, et al. Assessment of visuo-attentional abilities in young children with or without visual disorder: toward a systematic screening in the general population. Res Dev Disabil [Internet]. 2010 Sep [cited 2022 Nov 9];31(5):1102–8. Available from: https://pubmed.ncbi.nlm.nih.gov/20413265/

73. Ko J. Sensitivity to functional improvements of GMFM-88, GMFM-66, and PEDI mobility scores in young children with cerebral palsy. Percept Mot Skills. 2014/08/26. 2014;119(1):305–19.

74. Scott K, Lewis J, Pan X, Heathcock J. Parent-Reported PEDI-CAT Mobility and Gross Motor Function in Infants With Cerebral Palsy. Pediatr Phys Ther [Internet]. 2021/06/05. 2021;33(3):156–61. Available from: https://www.ingentaconnect.com/content/wk/pep/2021/00000033/00000003/art00013;jsessionid=3kiit2ff2cua.x-ic-live-03

For peer review only

FIGURE

Figure 1: CONSORT Flowchart. CP, cerebral palsy; HABIT-ILE, Hand and Arm Bimanual Intensive Therapy Including Lower Extremities.

For peer review only

For peer review only - http://bmjopen.bmj.com/site/about/guidelines.xhtml

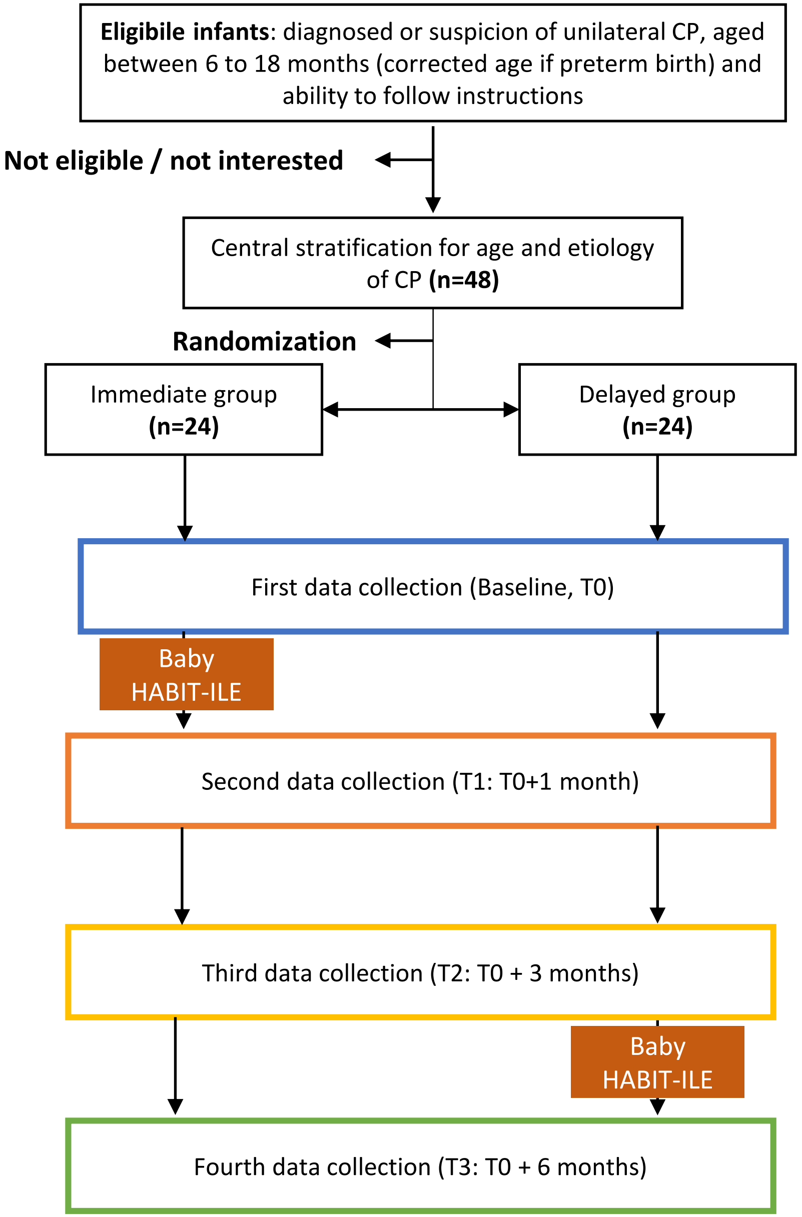

Assessments of T0, T1, T2 & T3

Body functions and structures

- Ophthalmological assessments (only once)
- Neuroimaging (MRI): anatomical imaging (T1, T2 and multi-shell diffusion) & functional imaging (resting-state)

Activity

- Mini-Assisting Hand Assessment (Mini-AHA; main outcome)
- Gross Motor Function Measure – 66 (GMFM-66)
- The Bayley Scales of Infant and toddler Development – Third Edition (BSID-III): Gross and fine motor, receptive and expressive communication & cognitive scales
- Kinematics of the upper extremity
- « Batterie d'évaluation du Jeune Enfant" (BAJE)
- The Pediatric Evaluation of Disability Inventory - computer adaptive test (PEDI-CAT)
- Canadian Occupational Performance Measure (COPM)

Participation

- The Pediatric Evaluation of Disability Inventory - computer adaptive test (PEDI-CAT)
- Young Children's Participation and Environment Measure (YCPEM)
- Canadian Occupational Performance Measure (COPM)

Reporting checklist for protocol of a clinical trial.

Based on the SPIRIT guidelines.

Instructions to authors

Complete this checklist by entering the page numbers from your manuscript where readers will find each of the items listed below.

Your article may not currently address all the items on the checklist. Please modify your text to include the missing information. If you are certain that an item does not apply, please write "n/a" and provide a short explanation.

Upload your completed checklist as an extra file when you submit to a journal.

In your methods section, say that you used the SPIRITreporting guidelines, and cite them as:

Chan A-W, Tetzlaff JM, Gøtzsche PC, Altman DG, Mann H, Berlin J, Dickersin K, Hróbjartsson A, Schulz KF, Parulekar WR, Krleža-Jerić K, Laupacis A, Moher D. SPIRIT 2013 Explanation and Elaboration: Guidance for protocols of clinical trials. BMJ. 2013;346:e7586

		Reporting Item	Page Number
**Administrative information**			
Title	#1	Descriptive title identifying the study design, population, interventions, and, if applicable, trial acronym	1
Trial registration	#2a	Trial identifier and registry name. If not yet registered, name of intended registry	2,15
Trial registration: data set	#2b	All items from the World Health Organization Trial Registration Data Set	n/a
Protocol version	#3	Date and version identifier	n/a
Funding	#4	Sources and types of financial, material, and other support	17
Roles and responsibilities: contributorship	#5a	Names, affiliations, and roles of protocol contributors	1,16,17

For peer review only

Roles and responsibilities: sponsor contact information	#5b	Name and contact information for the trial sponsor	n/a
Roles and responsibilities: sponsor and funder	#5c	Role of study sponsor and funders, if any, in study design; collection, management, analysis, and interpretation of data; writing of the report; and the decision to submit the report for publication, including whether they will have ultimate authority over any of these activities	17
Roles and responsibilities: committees	#5d	Composition, roles, and responsibilities of the coordinating centre, steering committee, endpoint adjudication committee, data management team, and other individuals or groups overseeing the trial, if applicable (see Item 21a for data monitoring committee)	n/a
**Introduction**			
Background and rationale	#6a	Description of research question and justification for undertaking the trial, including summary of relevant studies (published and unpublished) examining benefits and harms for each intervention	4,5
Background and rationale: choice of comparators	#6b	Explanation for choice of comparators	4,5
Objectives	#7	Specific objectives or hypotheses	6
Trial design	#8	Description of trial design including type of trial (eg, parallel group, crossover, factorial, single group), allocation ratio, and framework (eg, superiority, equivalence, non-inferiority, exploratory)	6
**Methods: Participants, interventions, and outcomes**			
Study setting	#9	Description of study settings (eg, community clinic, academic hospital) and list of countries where data will be collected. Reference to where list of study sites can be obtained	6
Eligibility criteria	#10	Inclusion and exclusion criteria for participants. If applicable, eligibility criteria for study centres and individuals who will	7

For peer review only

		perform the interventions (eg, surgeons, psychotherapists)	
Interventions: description	#11a	Interventions for each group with sufficient detail to allow replication, including how and when they will be administered	9, 10
Interventions: modifications	#11b	Criteria for discontinuing or modifying allocated interventions for a given trial participant (eg, drug dose change in response to harms, participant request, or improving / worsening disease)	n/a
Interventions: adherance	#11c	Strategies to improve adherence to intervention protocols, and any procedures for monitoring adherence (eg, drug tablet return; laboratory tests)	9,10
Interventions: concomitant care	#11d	Relevant concomitant care and interventions that are permitted or prohibited during the trial	n/a
Outcomes	#12	Primary, secondary, and other outcomes, including the specific measurement variable (eg, systolic blood pressure), analysis metric (eg, change from baseline, final value, time to event), method of aggregation (eg, median, proportion), and time point for each outcome. Explanation of the clinical relevance of chosen efficacy and harm outcomes is strongly recommended	10-15
Participant timeline	#13	Time schedule of enrolment, interventions (including any run-ins and washouts), assessments, and visits for participants. A schematic diagram is highly recommended (see Figure)	6
Sample size	#14	Estimated number of participants needed to achieve study objectives and how it was determined, including clinical and statistical assumptions supporting any sample size calculations	7,8
Recruitment	#15	Strategies for achieving adequate participant enrolment to reach target sample size	7
**Methods: Assignment of interventions (for controlled trials)**			
Allocation: sequence generation	#16a	Method of generating the allocation sequence (eg, computer-generated random numbers), and list of any factors for stratification. To reduce predictability of a random sequence, details of any planned restriction (eg, blocking) should be provided in a separate document that is unavailable to those who enrol participants or assign interventions	8

For peer review only

Allocation concealment mechanism	#16b	Mechanism of implementing the allocation sequence (eg, central telephone; sequentially numbered, opaque, sealed envelopes), describing any steps to conceal the sequence until interventions are assigned	n/a
Allocation: implementation	#16c	Who will generate the allocation sequence, who will enrol participants, and who will assign participants to interventions	8,17
Blinding (masking)	#17a	Who will be blinded after assignment to interventions (eg, trial participants, care providers, outcome assessors, data analysts), and how	8
Blinding (masking): emergency unblinding	#17b	If blinded, circumstances under which unblinding is permissible, and procedure for revealing a participant's allocated intervention during the trial	n/a
**Methods: Data collection, management, and analysis**			
Data collection plan	#18a	Plans for assessment and collection of outcome, baseline, and other trial data, including any related processes to promote data quality (eg, duplicate measurements, training of assessors) and a description of study instruments (eg, questionnaires, laboratory tests) along with their reliability and validity, if known. Reference to where data collection forms can be found, if not in the protocol	10
Data collection plan: retention	#18b	Plans to promote participant retention and complete follow-up, including list of any outcome data to be collected for participants who discontinue or deviate from intervention protocols	n/a
Data management	#19	Plans for data entry, coding, security, and storage, including any related processes to promote data quality (eg, double data entry; range checks for data values). Reference to where details of data management procedures can be found, if not in the protocol	10
Statistics: outcomes	#20a	Statistical methods for analysing primary and secondary outcomes. Reference to where other details of the statistical analysis plan can be found, if not in the protocol	15
Statistics: additional analyses	#20b	Methods for any additional analyses (eg, subgroup and adjusted analyses)	n/a

For peer review only

Statistics: analysis population and missing data	#20c	Definition of analysis population relating to protocol non-adherence (eg, as randomised analysis), and any statistical methods to handle missing data (eg, multiple imputation)	n/a
**Methods: Monitoring**			
Data monitoring: formal committee	#21a	Composition of data monitoring committee (DMC); summary of its role and reporting structure; statement of whether it is independent from the sponsor and competing interests; and reference to where further details about its charter can be found, if not in the protocol. Alternatively, an explanation of why a DMC is not needed	n/a
Data monitoring: interim analysis	#21b	Description of any interim analyses and stopping guidelines, including who will have access to these interim results and make the final decision to terminate the trial	n/a
Harms	#22	Plans for collecting, assessing, reporting, and managing solicited and spontaneously reported adverse events and other unintended effects of trial interventions or trial conduct	10
Auditing	#23	Frequency and procedures for auditing trial conduct, if any, and whether the process will be independent from investigators and the sponsor	n/a
**Ethics and dissemination**			
Research ethics approval	#24	Plans for seeking research ethics committee / institutional review board (REC / IRB) approval	15
Protocol amendments	#25	Plans for communicating important protocol modifications (eg, changes to eligibility criteria, outcomes, analyses) to relevant parties (eg, investigators, REC / IRBs, trial participants, trial registries, journals, regulators)	15
Consent or assent	#26a	Who will obtain informed consent or assent from potential trial participants or authorised surrogates, and how (see Item 32)	15
Consent or assent: ancillary studies	#26b	Additional consent provisions for collection and use of participant data and biological specimens in ancillary studies, if applicable	n/a
Confidentiality	#27	How personal information about potential and enrolled participants will be collected, shared, and maintained in order to	15

For peer review only

		protect confidentiality before, during, and after the trial	
Declaration of interests	#28	Financial and other competing interests for principal investigators for the overall trial and each study site	17
Data access	#29	Statement of who will have access to the final trial dataset, and disclosure of contractual agreements that limit such access for investigators	15
Ancillary and post trial care	#30	Provisions, if any, for ancillary and post-trial care, and for compensation to those who suffer harm from trial participation	n/a
Dissemination policy: trial results	#31a	Plans for investigators and sponsor to communicate trial results to participants, healthcare professionals, the public, and other relevant groups (eg, via publication, reporting in results databases, or other data sharing arrangements), including any publication restrictions	15
Dissemination policy: authorship	#31b	Authorship eligibility guidelines and any intended use of professional writers	n/a
Dissemination policy: reproducible research	#31c	Plans, if any, for granting public access to the full protocol, participant-level dataset, and statistical code	15
**Appendices**			
Informed consent materials	#32	Model consent form and other related documentation given to participants and authorised surrogates	appendix 1
Biological specimens	#33	Plans for collection, laboratory evaluation, and storage of biological specimens for genetic or molecular analysis in the current trial and for future use in ancillary studies, if applicable	n/a

The SPIRIT Explanation and Elaboration paper is distributed under the terms of the Creative Commons Attribution License CC-BY-NC. This checklist was completed on 17. July 2023 using https://www.goodreports.org/, a tool made by the EQUATOR Network in collaboration with Penelope.ai

For peer review only
